# DESE: estimating driver tissues by selective expression of genes associated with complex diseases or traits

**DOI:** 10.1186/s13059-019-1801-5

**Published:** 2019-11-06

**Authors:** Lin Jiang, Chao Xue, Sheng Dai, Shangzhen Chen, Peikai Chen, Pak Chung Sham, Haijun Wang, Miaoxin Li

**Affiliations:** 1 Zhongshan School of Medicine, Center for Precision Medicine, Sun Yat-sen University, Guangzhou, 510080 China; 2 Department of Pituitary Tumour Center, The First Affiliated Hospital, Sun Yat-sen University, Guangzhou, 510080 China; 3 Key Laboratory of Tropical Disease Control (SYSU), Ministry of Education, Guangzhou, 510080 China; 4 Department of Psychiatry, The Centre for Genomic Sciences, State Key Laboratory of Brain and Cognitive Sciences, The University of Hong Kong, Hong Kong SAR, China

**Keywords:** Tissue-selective expression, Disease driver-tissues, Susceptibility genes, Gene-based association, Genome-wide association study

## Abstract

The driver tissues or cell types in which susceptibility genes initiate diseases remain elusive. We develop a unified framework to detect the causal tissues of complex diseases or traits according to selective expression of disease-associated genes in genome-wide association studies (GWASs). This framework consists of three components which run iteratively to produce a converged prioritization list of driver tissues. Additionally, this framework also outputs a list of prioritized genes as a byproduct. We apply the framework to six representative complex diseases or traits with GWAS summary statistics, which leads to the estimation of the lung as an associated tissue of rheumatoid arthritis.

**Electronic supplementary material:**

The online version of this article (10.1186/s13059-019-1801-5) contains supplementary material, which is available to authorized users.

## Background

Tissue selectivity is an important characteristic of many complex diseases or traits [1]. A complex phenotype often involves multiple related tissues, some of which are implicit [2]. Unfortunately, our current knowledge on the causal tissues of complex diseases is often limited in clinical observations. For example, it is certain that the brain must be a relevant organ of schizophrenia. However, as human brains consist of multiple heterogeneous regions, it is crucial to know which regions are the actual drivers [3]. Human height is another typical example. It is generally known that cell proliferation in multiple tissues (e.g., skeletal and cardiac muscle) may contribute to the development of human height [4]. But it is unclear which tissues are primarily more important for the development of height. For most of human diseases and traits, the primary driver tissues remain elusive [5].

The pathology of tissue selective may be attributed to the selective expression of their susceptibility genes [6, 7]. Many studies showed that disease causal genes tend to have elevated selective expression in the pathogenic tissues [1, 6], implicating a basis for the tissue selectivity of diseases. Analyses of genes’ selective expression profiles can expand the knowledge on human diseases [8] and even can facilitate characterizing new causal genes [9]. Recently, Ongen et al. proposed to estimate the causal tissues for complex traits and diseases by measuring the genome-wide association study (GWAS)-associated variants’ eQTL activity in different tissues [10]. Finucane et al. also developed a method to estimate disease-relevant tissues according to heritability enrichment in specifically expressed genes by linkage-disequilibrium (LD) score regression approach [11]. But neither of the methods directly employs the quantity of genes’ selectivity expression for driver-tissue estimation nor do they directly characterize susceptibility genes based on the estimation.

Tissue-selective expression refers to much higher or lower expression of a gene in one or some minority tissues compared to majority tissues [12]. However, it is difficult to quantify the relative difference due to ambiguous boundaries between the minority and the majority in practice. There have been several methods for detecting tissue-selective expression of genes (see method description in the review [13]). Most early methods are omnibus tests and can only tell whether a gene has overall selective expression [14, 15], and most recent methods are underpowered to detect selective expression at individual tissues when there is more than one tissue with selective expression [16]. Meanwhile, these ever-increasing transcriptomic resources [17–21] (including GTEx) are calling for more powerful selective expression measures and more studies on tissue-selective pathology of human diseases.

In this study, we proposed a unified framework to estimate driver tissues or cell types of complex diseases or traits based on selective expression of phenotype-associated genes of GWAS. After investigating the selective expression in GTEx Project [20] by a new measure, we further applied this framework to identify potential driver tissues of six representative complex phenotypes with GWAS summary statistics and investigated how the prioritized tissues can help enhance detection of susceptibility genes in secondary analyses of GWAS data. For simplicity, being tissue selective means being tissue- or cell-type selective throughout the paper.

## Results

### The proposed framework of estimating driver tissues and its robust *z*-score for selective expression

**Fig. 1:**
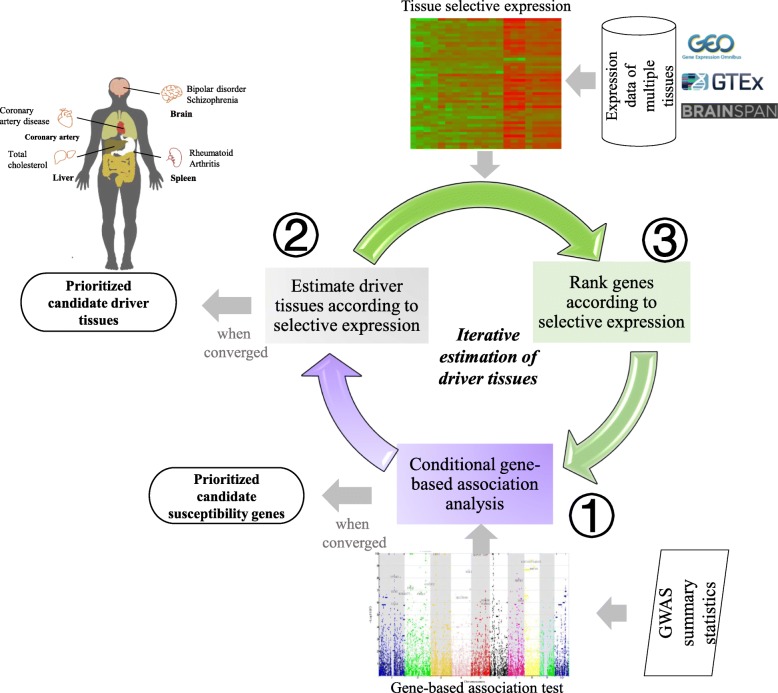
The diagram of the unified framework. The unified framework, DESE, consists of three components which run iteratively. The iteration converged until the *p* values of estimated tissues get stable. DESE needs two input datasets, the tissue-selective expression profiles and GWAS summary statistics. It outputs estimated driver tissues and prioritized genes

The framework, named driver-tissue estimation by selective expression (DESE), aims to estimate driver tissues by tissue-selective expression of phenotype-associated genes in GWAS (see the workflow in Fig. 1). The assumption is that the tissue-selective expression of causal or susceptibility genes indicates the tissues where complex phenotypes happen primarily [1], which are called driver or causal tissues. Therefore, a driver tissue is very likely to be enriched with selective expression of susceptibility genes of a phenotype. The framework requires two types of input data, gene expression values of multiple tissues and GWAS summary statistics or association *p* values at variants for a tested phenotype. The expression values at genes and transcripts or even exons can be used for the estimation. The GWAS *p* values are used to detect susceptibility genes by a conditional gene-based association test we published recently [22]. The framework has three components running iteratively and converges when statistical *p* values of estimated driver tissues become stable (see the workflow in Fig. 1). A byproduct of the framework is a list of prioritized genes which have both significant selective expression in the estimated driver tissues and significant conditional gene-based *p* values for the tested diseases or traits. DESE has been implemented into our platform KGG (see the graphic interface in Additional file 1: Figure S8), http://grass.cgs.hku.hk/limx/kgg/.

A critical datatype of the unified estimation framework is the tissue-selective expression. We proposed a measure (named robust-regression *z*-score) of selective expression by extending the Huber robust linear regression. The method fits a robust line for ranked expression values of genes to calculate expression deviation and integrates expression variation to measure selective expression (see details in the “Methods” section). Under null hypothesis, it produces *p* values approximately under uniform distribution (see the QQ plots in Additional file 1: Figures S2 and S3), which will greatly facilitate statistical inference of selective expression. Extensive computer simulations show that the robust-regression *z*-score is more powerful than the conventional *z*-score when there are multiple selectively expressed tissues (Additional file 1: Table S1). We also provide a webserver for an online query of the robust selective expression of genes in different tissues or cell types, http://grass.cgs.hku.hk/limx/rez/.

### Tissue-selective expression profiles in 50 tissues produced by the robust-regression *z*-score

**Fig. 2:**
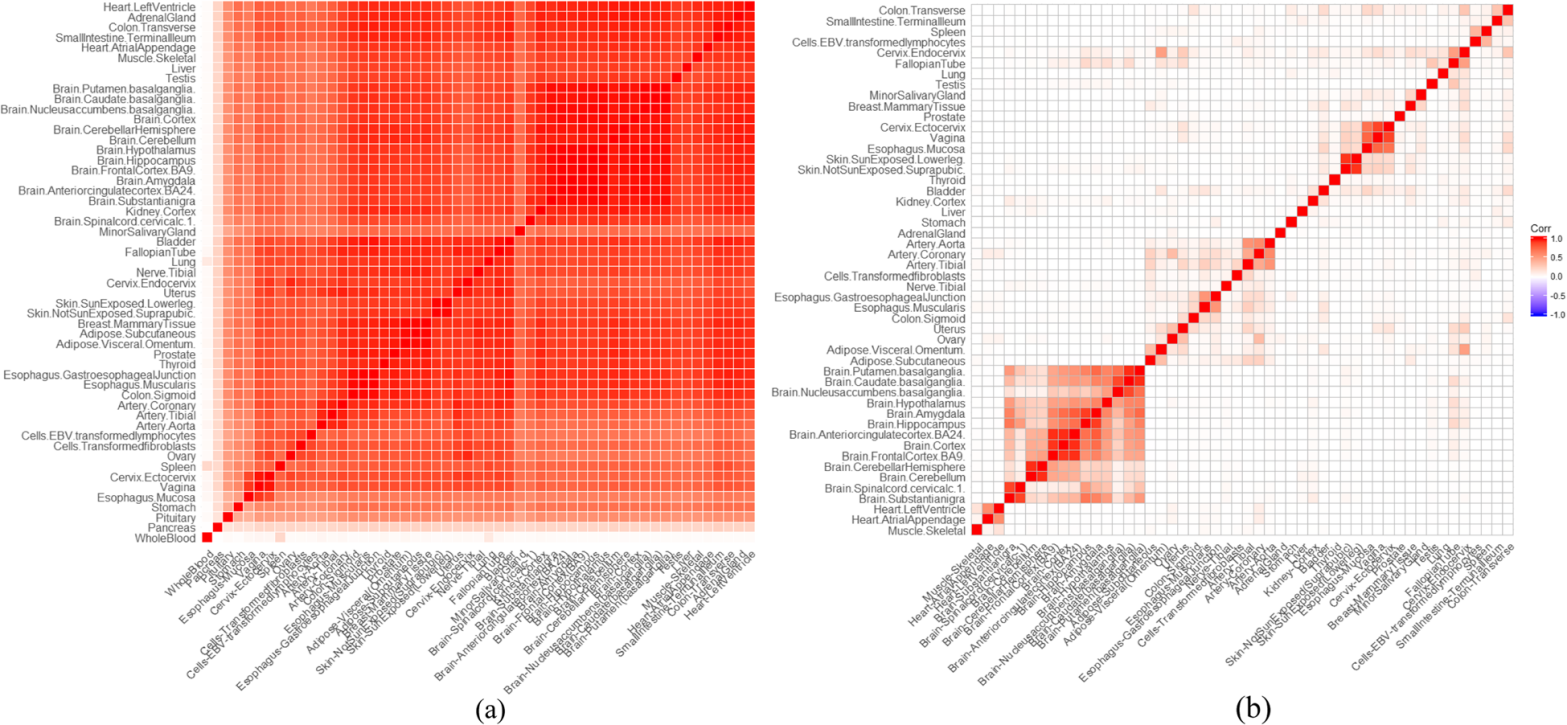
Pearson correlation of the tissues by original expression and selective expression. **a** The correlation by original TPM expression values at the transcript level. **b** The correlation by selective expression measured by the robust-regression *z*-score expression at the transcript level

The robust-regression *z*-score approach was applied to generate tissue-selective expression profiles by using RNA-Seq data from GTEx project (V7) [20] after stringent quality control (see details in the “Methods” section). While the number of selectively expressed genes varied from tissues to tissues (Additional file 1: Table S5), the profiles had three common interesting patterns. First, the pair-wise Pearson correlations between tissues based on the robust-regression *z*-scores were substantially different from those based on the original expression values by transcripts per million (TPM) (Fig. 2). Most tissue pairs had the correlation over 0.7 (Pearson coefficients) based on the expression values (Fig. 2a and Additional file 1: Figure S4) while most of them had zero correlation based on the robust-regression *z*-score (Fig. 2b and Additional file 1: Figure S5). However, tissues of similar origins had high Pearson correlations (> 0.8) of the robust-regression *z*-scores, such as Skin-SunExposed (Lowerleg) vs. Skin-NotSunExposed (Suprapubic) pair. The biologically sensible consistency suggested the robust-regression *z*-score quantified tissue-selective expression of genes correctly. Second, the original expression values of a tissue had low correlation with the selective expression values of the same tissue. As shown in Additional file 1: Figures S6 and S7, only 12 tissues have moderate Spearman correlation coefficient, *r*∈[0.3, 0.6] between the original expression values and selective expression values at gene level. Most tissues had nearly zero or even negative correlations. This observation means high expression does not necessarily mean high selective expression. Third, the expression data at the transcript level led to the discovery of much more selectively expressed genes. In most tissues, the usage of transcript-level expression detected on average 54% extra selectively expressed genes which were missed by the usage of gene-level expression (Additional file 1: Table S5). Although the conservative Bonferroni correction for multiple transcripts of a gene may lead to the missing of some selectively expressed genes, the unique genes selectively expressed according to transcript-level expression were still on average 5.5 times more than that according to gene-level expression in the 50 tested tissues.

### Estimate driver tissues in six complex diseases/traits

Based on the selective expression, we then applied DESE to estimate driver tissues with the usage of public available GWAS summary statistics. Six representative complex diseases/traits were used as proof-of-principle examples.

#### Schizophrenia

**Fig. 3:**
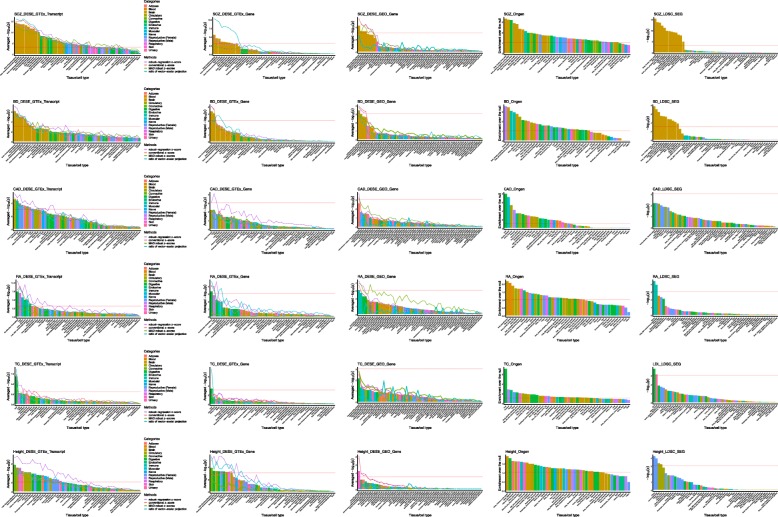
Driver tissues estimated by DESE and two exiting methods in six representative complex diseases/traits. Note: Each row shows one disease/trait. The first, second, and third columns show the estimated driver tissues according to GTEx transcript-level, GTEx gene-level, and GEO gene-level selective expression respectively. The bar denotes the averaged -log_10_(*p*) based on selective expression of four different measures. The -log_10_(*p*) based on each selective expression measure is denoted by a line. The fourth column shows estimated driver tissues by Ongen et al.’s method, which is extracted from Supplementary Table 5 of their published paper [10]. The fifth column shows estimated driver tissues by the LDSC-SEG method, which is extracted from Supplementary Table 6 of their published paper [11]. The pink horizontal denotes the significance level. The tissues are classified into 15 groups according to anatomy. The tissues are sorted by the averaged -log_10_(*p*) on *y* axis in descending order. SCZ schizophrenia, BD bipolar disorder, CAD coronary artery disease, RA rheumatoid arthritis, TC total cholesterol

We used *p* values from a large-scale Meta-GWAS study [23] to estimate driver tissues of schizophrenia by DESE with selective expression. While consistent with the known biology that the top 10 tissues are all brain regions (see details in Fig. 3), there were several interesting points in the results. First, the statistical significance of estimated driver tissues based on transcript-level selective expression was much higher than that based on gene-level selective expression. For example, the *p* value of the top tissue according to the transcript-level selective expression by the robust-regression *z*-score was 5.3E−13 while that according to gene-level selective expression was only 2.0E−5. This pattern was also true when selective expression was measured by three alternative methods, conventional *z*-score, MAD robust *z*-score, and ratio of vector-scalar projection. Second, the ranking order of the prioritized tissues by the four different measures of selective expression was similar generally although the significance level varied. With the gene-level selective expression, the ratio of vector-scalar projection measure led to the highest significance level as small as 1E−10. The most significant tissue by the proposed robust-regression *z*-score and conventional *z*-score only achieved the *p* value 2.0E−5 and 4.8E−5 respectively. The robust *z*-score by MAD achieved the lowest significance level. With the transcript-level selective expression, the pattern was similar in which the ratio of vector-scalar projection measure achieved the highest significance level among the four measures. Because, however, there were also fluctuations in the ranking order according to different selective expression measures, we produced a combined ranking by averaging the -log(*p*) of the four selective expression measures. The top 2 estimated driver tissues according to both gene and transcript levels of selective expression were the frontal cortex and anterior cingulate cortex. It has been widely accepted that the frontal cortex [24] and anterior cingulate cortex [25] are critical brain regions for schizophrenia. Meanwhile, it should also be noted that most brain regions (e.g., nucleus accumbens and amygdala) are significant. A quick literature search in the NCBI PubMed database showed these estimated brain regions were supported by numerous published papers, which suggests DESE produced correct and consistent results for this complex brain disorder.

#### Bipolar disorder (BD)

For bipolar disorder, we used the summary statistics from a GWAS dataset containing 20,129 BD cases and 54,065 control cases [26]. Again, the transcript-level selective expression led to higher statistical significance than the gene-level selective expression (Fig. 3). With the transcript-level selective expression, the proposed robust-regression *z*-score produced the smallest *p* value among the four selective expression methods. However, probably due to fewer genuine susceptibility genes in this GWAS with smaller sample size, the statistical significance of prioritized tissues was less than that of schizophrenia in general. The top estimated driver tissues based on the transcript- and gene-level selective expression were the cerebellar hemisphere (*p* = 1.3E−09 and 9.0E−06 respectively according to the robust-regression *z*-score). There have been many studies implicating the cerebellum as a causal region of BD (e.g., [27–29]. The frontal cortex and anterior cingulate cortex were ranked as the second and third driver brain regions, at which the transcript-level selective expression by the robust-regression *z*-score led to significant *p* values (1.6E−6 and 2.4E−5). Note the two regions were ranked as the top driver brain regions of schizophrenia in above analysis. The common driver brain region is consistent with the high comorbidity and common genetic determinants of the two brain disorders [30]. Besides the three significant brain regions, there were also several other significant regions for BD, including the hypothalamus [31] and basal ganglia [32]. The consistency of the estimated brain tissues and known biology of this brain disease suggests the effectiveness of DESE.

#### Coronary artery disease (CAD)

Coronary artery disease causes impaired blood flow in the arteries that deliver blood from the heart to other body parts [33]. As expected, the coronary artery was reported as the top tissue according to the combined ranking at both gene and transcript levels (see details in Fig. 3). Meanwhile, the transcript-level selective expression led to a higher statistical significance than the gene-level (4.3E−6 vs. 9E−4) by the robust-regression *z*-score at the coronary artery. We also found that the aorta and tibial artery had significant *p* values (1.4E−4 and 6.1E−4) for CAD, probably because CAD-associated genes were also selectively expressed among different types of artery. It should be noted that the adipose tissue was prioritized as the fifth driver tissue with significant *p* value (5E−5 according to the proposed robust-regression *z*-score) by transcript-level selective expression, which is consistent with the studies suggesting the relevance between adipose tissues and CAD [34–36]. Golia et al. reviewed the role of adipocytokines as a possible link between obesity and vascular disease recently [34], suggesting the possible pathogenic mechanisms of adipose tissues in CAD. Interestingly, some of the female reproductive organs [such as the ovary (*p* = 6.1E−6) and uterus (*p* = 5.2E−5)] and adrenal gland (*p* = 1.7E-5) were also estimated as significant driver tissues of CAD. Although epidemiologic studies have reported the relatedness between ovary disease, uterine disease, adrenal insufficiency and CAD, or other cardiovascular diseases respectively [37–39], the underlying genetic mechanism has not been clear yet. The estimated causal tissues of CAD may shed some insights into the mechanism.

#### Rheumatoid arthritis (RA)

RA is a common autoimmune disease mainly attacking the joints [40]. Consistent with the pathology of autoimmune disease, two tissues among the top 5 driver tissues fell into the immune system (Fig. 3), spleen and lymphocytes (*p* = 7E−8 and 1.3E−6 respectively according to the proposed robust-regression *z*-score at the transcript level). As a non-immune tissue, the lung was prioritized as the second significant driver tissue (*p* = 4.2E−9) according to the robust-regression *z*-score at the transcript level. Demoruelle et al. recently reviewed the role of the lung in the pathogenesis of RA [41]. Multiple studies have identified a high prevalence of lung disease, both airways and parenchymal disease, in subjects with clinically classifiable RA. Demoruelle et al. proposed a model of the role of the lung in pathogenesis of RA, which basically suggested the RA-related autoantibodies may be initiated from lung autoimmuneresponse [41]. While confirming the strong associations between the lung and RA, the selective expression of RA associated genes in lung from our analysis may also provide new insights into the underlying mechanism. In addition, the ileum and transverse colon had significant *p* values for RA (5.5E−11 and 5.2E−10 respectively according to the robust-regression *z*-score at the transcript level), which is also consistent with the previous study showing the high prevalence of ileocolonic abnormality in RA [42].

#### Total cholesterol (TC)

For a complex clinical trait, TC, our analysis detected the liver as the most significant tissue. This is consistent with known knowledge that the liver is responsible for 80% of the endogenous cholesterol synthesis. We noticed that the significance level of the second tissue decreased substantially (*p* = 6.9E−8 vs. 3.3E−5 according to the proposed robust-regression *z*-score at the transcript level, see details in Fig. 3), which suggests that the liver is the major driver tissue for total cholesterol. The lung and spleen were also estimated as significant driver tissues (*p* = 3.3E−5 and 3.5E−5 respectively according to the robust-regression *z*-score at the transcript level) and have been shown to be involved in lipid metabolism [43, 44].

#### Height

For the anthropometric trait, height, 27 significant tissues (*p* < 10^−3^) were detected according to the selective expression by the robust-regression *z*-score at the transcript level (see details in Fig. 3), which implies complex biological mechanisms in the development of human height. The most significant tissue was fibroblast, which is the most common cell type of connective tissue in mammals. Consistent with our results, several studies have also reported connective tissue as the most enriched tissue type for height [45, 46]. Besides the fibroblast cell, the top 10 driver tissues include the cardiovascular tissues (i.e., heart-atrialappendag, artery-coronary, artery-aorta, artery-tibial), esophagus, adipose, lung, and uterus, which may provide some new insights into the mechanism of height.

### Validate estimated driver tissues by existing tools

We first validated the above estimated driver tissues with two existing tools, Ongen et al. [10] and LDSC-SEG [11]. The two tools estimated driver tissues based on different techniques (see details in the “Methods” section). We assume that the driver tissues replicated by independent approaches are more likely to be true. Figure 3 visualizes the validation and comparison results of the three tools.

#### Schizophrenia

Among the top 10 significant driver tissues of schizophrenia by Ongen et al., six were sub-brain regions. They are brain frontal cortex_BA9, brain anterior cingulate cortex_BA24, brain putamen basal ganglia, brain hippocampus, brain cerebellar hemisphere, and brain hypothalamus. Five out of the six (except for the brain cerebellar hemisphere) were also among the top 10 estimated significant driver tissues by DESE. Interestingly both tools ranked the brain frontal cortex (BA9) as the top driver tissue of schizophrenia. Consistent with DESE, all the top 10 estimated driver tissues by LDSC-SEG were brain regions. Three of the top 5 estimated brain regions by the two different tools were overlapped (brain frontal cortex-BA9, brain anterior cingulate cortex-BA24, and brain cortex), in which the brain frontal cortex-BA9 was also ranked as the top driver tissue by LDSC-SEG. The contribution of the frontal cortex and the other brain regions to schizophrenia was also successfully validated with tissue-specific chromatin data in the paper introducing LDSC-SEG (see details in Supp Table 7 of [11]). The high consistency between the results by DESE and that of the other tools clearly confirmed the validity of DESE for driver-tissue estimation. Moreover, DESE outperformed Ongen et al. in terms of false positive finding.

#### Bipolar disorder (BD)

Estimation of driver tissues for BD seemed more difficult than that for schizophrenia. Among the top 10 estimated significant driver tissues by Ongen et al., only two were sub-brain regions, brain anterior cingulate cortex (BA24) and hippocampus, for this brain disorder. Both of the sub-brain regions were also significant, *p* = 3.3E-5 and 1.8E-3 (suggestively), according to the *p* values by DESE with the transcript-level selective expression. The top significant driver tissue by Ongen at al.’s approach was prostate, which we failed to verify through a literature survey. The results of DESE and LDSC-SEG were much more similar. The top 8 estimated driver tissues by both tools were all brain regions. There were three common brain regions among the top 5 estimated driver regions by the two tools, brain frontal cortex (BA9), brain anterior cingulate cortex (BA24), and brain cortex. As stated above, these tissues were implicated with BD by many studies. The top driver tissue by DESE, cerebellum, was also significant by LDSC-SEG (*p* = 1.9E−5) although it was not the top tissue by the latter. Therefore, DESE’s estimated driver tissues for BD were highly constant with that estimated by LDSC-SEG and were concordant with known biology of this brain disease.

#### Coronary artery disease (CAD)

The top significant tissue by DESE, the coronary artery, was estimated as the third and eighth significant tissues for CAD by Ongen et al.’s approach and LDSC-SEG respectively. Consistent with our results, the female reproductive organs (such as ovary, cervix and uterus) prioritized by DESE were detected as the top 5 significant tissues by LDSE-SEG, and the adrenal gland prioritized by DESE was detected as the top 5 significant tissue by Ongen et al.’s approach. However, the adipose tissue estimated as the driver tissue by DESE was not detected among the top 10 tissues by the two existing approaches. LDSC-SEG estimated the ileum as the most significant tissue, and we failed to find literatures supporting this. The liver was detected as the top significant tissue for CAD by Ongen et al.’s approach but failed by LDSC-SEG and DESE. Therefore, DESE produced not only consistent driver tissues with existing tools but also extra promising driver tissues of CAD.

#### Rheumatoid arthritis (RA)

The immune tissues, spleen and lymphocytes, were prioritized in the top five driver tissues by DESE, which were supported by the similar findings from LDSC-SEG. Ongen et al. prioritized spleen as the tenth significant driver tissue, the only immune tissue in the top 10. Unexpectedly, it estimated the brain region hypothalamus as the most significant tissues. We only found subtle alterations in hypothalamic-pituitary-adrenal system axis mainly affect the adrenal level [47]. Hence, the effect of hypothalamic in RA is unclear.

#### Total cholesterol (TC)

Due to lack of TC results in LDSC-SEG study, we selected the similar trait LDL (low-density lipoproteins) for comparison in this part. The liver was also detected as the most significant tissue for TC (or LDL) by both LDSC-SEG and Ongen et al.’s approach, which is consistent with the fact that liver contributes to most of lipoprotein metabolism [48]. Similarly, the significance of the liver by Ongen et al.’s approach was much higher than the subsequent tissues, such as the second tissue pancreas (7.0 vs. 2.8 according to the enrichment over the null). Therefore, the strong consistence of the three approaches and pathological knowledge confirmed the validity of DESE for estimation of driver tissues of clinical traits.

#### Height

Cardiovascular tissues for height by DESE were almost perfectly validated among the top 10 significant tissues by both Ongen et al.’s approach and LDSC-SEG (Artery_Coronary, Artery_Aorta by Ongen et al.’s approach; Artery_Tibial, Artery_Coronary, Artery_Aorta by LDSC-SEG), suggesting the important role of cardiovascular tissues in height. Moreover, both LDSC-SEG and DESE detected the connective tissues (i.e., transformed fibroblasts) among the top tissues, which is consistent with previous studies [45, 46] as described above. However, Ongen et al.’s approach detected several brain tissues (such as basal ganglia, cerebellar hemisphere) in the top 10 tissues, which was supported by an association study [49]. Unexpectedly, LDSC-SEG estimated two female reproductive tissues (uterus and endocervix) as the top 2 tissues, but we failed to find supporting literatures.

### Validate estimated driver tissues with independent expression data

Besides the above validation at technique level, we also carried out validation at data level. The validation was performed by microarray data of 55 tissues curated with stringent quality control from the GEO database (see details in the “Methods” section).

#### Schizophrenia

Consistent with the above results based on the RNA-Seq expression from GTEx project, all the top 8 driver tissues by DESE with the GEO dataset for schizophrenia were parts of brain regions (Fig. 3). This is a simple system-level validation. Among the eight brain regions, two regions [prefrontal-cortex (*p* = 7.2E−5) and hippocampus (*p* = 3.4E−5)] were exactly matched with the GTEx brain regions. Their *p* values according to the GTEx data by the same selective expression measure (the proposed robust-regression *z*-score) were also highly significant based on the transcript-level selective expression, *p* = 1.5E−14 and 9.4E−12, respectively. Note this was also a successful validation with different data types (RNA-Seq vs. microarray data) and independent samples. There have been numerous studies implicating the contribution of the two brain regions to schizophrenia [50, 51].

#### Bipolar disorder (BD)

For another brain disorder, BD had only one significant driver tissue, brain parietal lobe (*p* = 1.9E−4 based on selective expression by the robust-regression *z*-score) according to the GEO expression data. However, all the three subsequent suggestively significant driver tissues were also parts of the brain tissues, superior frontal gyrus, cerebral gray matter, and cerebral gyrus. Unfortunately, except for the cerebral gray matter, three of the other tissues had no matched region in the GTEx dataset. According to the structure of BRENDA tissue, the cerebral gray matter (BTO_0000823) is a part of the spinal cord. The *p* value of brain-spinal cord (cervicalc-1) based on the transcript-level selective expression from GTEx was significant, *p* = 3E−4. Anyhow, the top driver tissue based on the GEO data, parietal lobe, responsible for cognition (including attention and memory), has been implicated with BD by many studies [52].

#### Coronary artery disease (CAD)

The adipose tissue and two cardiovascular tissues (myocardium and left ventricle) were estimated among the top 4 driver tissues for CAD based on the GEO expression (*p* = 2.3E−4, 4.5E−2, and 3.2E−2 respectively by the proposed robust-regression *z*-score), which was consistent with the results based on GTEx dataset. Interestingly, the female reproductive tissue uterus was also replicated in the GEO dataset. Moreover, another female reproductive cell type oocyte was estimated as the second top tissues based on GEO dataset although its *p* value was no longer significant (*p* = 2.1E−2 according to the proposed robust-regression *z*-score). The high consistency of estimated driver tissues (including the female reproductive tissues) with independent sample successfully validated the results of DESE for CAD.

#### Rheumatoid arthritis (RA)

Consistent with results based on GTEx dataset, two immune tissues (tonsil and lymph node) were estimated as top 2 driver tissues based on GEO dataset (*p* = 7.2E−5 and 2.4E−4 respectively by the robust-regression *z*-score). Following the immune tissues, the colon and ileum (*p* = 3E−4 and 2.4E−3 respectively) were detected as the third and fourth driver tissues respectively, which were also consistent with the results based on GTEx. Due to lacking of expression data of the lung in GEO, we could not replicate the results of the lung. It should be noted that the blood was detected as driver tissues based on GEO dataset and also detected by LDSE-SEG while the GTEx whole blood was excluded in the QC procedure (see the “Methods” section).

#### Total cholesterol (TC)

Consistent with the results based on GTEx, the top tissue for TC based on GEO was hepatocyte (*p* value 3.4E−3 by the proposed robust-regression *z*-score). As we failed to collect sufficient number of expression profiles of lung and spleen in in GEO, their significant results in GTEx dataset cannot be validated. We also noticed that the significance of estimated tissues following hepatocyte was low, such as the second driver tissue with *p* value only 3.3E−2, which was also consistent with the pattern based on the GTEx data.

#### Height

Due to the tissue difference in GEO and GTEx dataset, we could not validate the results at the exact driver tissues but can do it at the system level. The top 2 driver tissues for height based on GEO data included two connective tissues knee and synovium with significant *p* values (1E−8 and 2.2E−4 respectively by the robust-regression *z*-score), which was consistent with the results based on GTEx dataset. Furthermore, the cardiovascular tissues (i.e., left ventricle), adipose, and uterus were also validated by GEO dataset with significant *p* values (5.7E−3, 1.5E−4, and 3.0E−4 respectively by the proposed robust-regression *z*-score) among the top 10 tissues.

### Fine estimation with brain-only expression data

For the brain disorders, it may be more interesting to finely prioritize the brain regions with expression only in the brain. We produced selective expression values among the 13 brain regions from GTEx and 16 brain regions from BrainSpan dataset and input them into DESE to estimate driver brain regions for schizophrenia and BD.

#### Schizophrenia

For schizophrenia, the top driver tissue remained the frontal cortex (BA9) (*p* = 3.3E−11 and 2.5E−7, based on the transcript-level and gene-level selective expression from GTEx by the robust-regression *z*-score) (see details at Additional file 2: Table S15 and S16). The ranking order was also similar to that based on the 50 GTEx tissues although there were some minor fluctuations. The major difference may be the significance level at the cerebellum. The *p* value at the cerebellum by DESE based on the 50 GTEx tissues was 1.1E−7 while that based on the 13 GTEx brain regions was only 0.015. We also validated the results with another independent brain dataset, BrainSpan. The significance at the frontal cortex was successfully replicated by both of the exon-level and gene-level selective expression at the orbital frontal cortex and ventrolateral prefrontal cortex (see details in Additional file 2: Table S17 and S18). Consistent with the results in the GTEx brain dataset, the cerebellar cortex was also ranked as the least significant region for schizophrenia. The significant brain regions inferred by the brain-only expression data may suggest a unique contribution of significant regions to this complex brain disorder.

#### Bipolar disorder (BD)

With the brain-only expression data, BD also showed several common significant driver tissues with those of schizophrenia. For examples, the top 2 driver regions of schizophrenia, frontal cortex (BA9) and anterior cingulate cortex (BA24), were also among the top 3 driver regions of BD (see details in Additional file 2: Table S19 and S20). The common regions also support the high comorbidity of the two brain disorders [53]. However, the two diseases also had unique estimated driver regions which may support different characteristics of the two diseases. The cerebellum, for example, was ranked as the top significant brain region of BD (*p* = 8.8E−6, based on the robust-regression *z*-score with transcript expression), while it had the lowest significance for schizophrenia (*p* = 0.015, based on the robust-regression *z*-score with transcript expression). The caudate nucleus was ranked as the fourth significant tissue for schizophrenia (*p* = 4.0E−8, based on the robust-regression *z*-score with transcript expression) while it was insignificant for BD (*p* = 0.1, based on the robust-regression *z*-score with transcript expression). Multiple regions were successfully replicated by the expression values from BrainSpan (see details in Additional file 2: Table S21 and S22). The orbital frontal cortex remained the top driver tissue of BD with the gene- and exon-level selective expression data from BrainSpan. The significance of the anterior cingulate cortex (BA24) with the GTEx data (*p* = 1.1E−5, based on the robust-regression *z*-score with transcript expression) was replicated by that of the anterior (rostral) cingulate (medial prefrontal) cortex from BrainSpan (*p* = 1.3E-3, based on the robust-regression *z*-score with gene-level).

### Investigate the contribution of lowly expressed genes

We noticed that lowly expressed genes also contributed to the prioritization of disease-related tissues when the transcript-level selective expression was used. A removal of lowly expressed genes led to substantial decrease in the statistical significance of estimated driver tissues at almost all the six complex phenotypes. As shown in Table 1, when minimal expression cutoffs were increased from 0.01 to 0.5 and 1.0 TPM for the GTEx data, the statistical significance of the top driver brain region of schizophrenia [frontal cortex (BA9)] was decreased from 5.3E−13 to 1.3E−07 and 1.5E−06 based on the transcript-level selective expression by the proposed robust-regression score. This was also true for other significant top brain regions although the ranking order of these brain regions was similar. The underlying cause is that a higher cutoff removed some genes that had relatively low expression but high selective expression. For example, CACNA1C is a well-known candidate susceptibility gene of schizophrenia [54]. CACNA1C encodes calcium voltage-gated channel subunit alpha1 C which is important for brain functions. This gene has 26 transcripts with expression in GTEx dataset, and 23 have very low expression. The transcript (ENST00000399641) had a large selective expression *z*-score 210.3 in the frontal cortex (*p* < 1.0E−200). However, it had only 0.2 TPM expression in the frontal cortex and nearly zero TPM in most tissues. A TPM cutoff even as low as 0.4 will exclude these important candidate genes of schizophrenia.

**Table 1:** The enrichment statistical significance for different minimal expression cutoffs

Cutoff	Schizophrenia			Bipolar disorder		
	Brain-anterior cingulate cortex (BA24)	Brain-frontal cortex (BA9)	Brain-cortex	Brain-cerebellar hemisphere	Brain-cerebellum	Brain-frontal cortex (BA9)
0.01	5.3E−13	5.3E−13	1.8E−12	1.3E−9	7.3E−09	1.6E−6
0.5	9.3E−8	1.3E−7	7E−8	1.7E−5	1.9E−5	6.4E−3
1.0	3.8E−7	1.5E−6	7.1E−7	3.0E−5	5.0E−5	0.024
	Coronary artery disease			Rheumatoid arthritis		
	Artery-coronary	Adrenal gland	Ovary	Small intestine-terminal ileum	Lung	Spleen
0.01	4.3E−6	1.7E−5	6.1E−6	5.5E−11	4.2E−9	7E−8
0.5	4.2E−4	8.9E−3	2.9E−5	6.3E−7	2.9E−8	6E−5
1.0	4.2E−3	4.7E−2	1.8E−4	1.2E−6	5.2E−8	1.7E−4
	Total cholesterol			Height		
	Liver	Lung	Spleen	Cells-transformed fibroblasts	Heart-atrial appendage	Lung
0.01	6.9E−8	3.3E−5	3.5E−5	1.3E−11	6E−12	5.3E−11
0.5	9.1E−5	1E−2	2.6E−2	1.9E−2	3.7E−3	6.6E−5
1.0	9.7E−3	6.4E−3	5.5E−2	4.3E−2	5.5E−3	1.5E−4

Note: The *p* values of driver tissues were calculated according to the proposed robust-regression *z*-scores. According to a cutoff *x*, gene or transcripts having TPM < *x* in 40 or more tissues were excluded

The nontrivial contribution of lowly expressed genes further suggested the selective expression may be more effective for driver-tissue estimation than original expression. In another experimental analysis, we also used the original expression for estimating the driver tissues by DESE to validate this argument. As shown in Additional file 1: Table S7, the *p* values of top driver tissues based on the original expression were much less significant than those based on the selective expression, around two orders of magnitude larger. Given that selective expression is different from expression (Additional file 1: Figures S6 and S7), selective expression (when available) instead of original expression may be preferable for prioritizing causal tissues and genes of phenotypes.

### Tissue selectivity prioritization enhances detection of susceptibility genes in post GWAS analyses

Finally, we asked how tissue selectivity in the estimated driver tissues can be used to enhance detection of genuine susceptibility genes in secondary analysis of GWAS data. The investigation was performed in the same six representative complex phenotypes as proof-of-principle examples.

#### Schizophrenia

In the schizophrenia dataset, the selective expression ranking led to ~ 32% significant genes which were not significant according to the disease-association *p* value ranking in the conditional gene-based test [22]. Among the different significant genes, a rough in silico validation in PubMed showed the selective expression ranking resulted in more genes implicated in schizophrenia by literature than the disease-association *p* value ranking (*n =* 6 vs. 0 with at least 9 supporting papers, see details in Additional file 2: Table S9). Here are some individual examples. In a set of physically close genes, the tissue-selective expression ranking and *p* value ranking led to two different significant genes, DRD2 and MIR4301, respectively. The DRD2 gene was selectively expressed in multiple prioritized pathogenic tissues of schizophrenia (including brain-anterior cingulate cortex, brain-cortex, brain-putamen (basal ganglia), and brain-spinal cord), and there were 100 papers co-mentioning the gene and schizophrenia in their titles or abstracts in PubMed database (see details in Additional file 2: Table S9). In contrast, there is no paper suggesting MIR4301’s contribution to schizophrenia. In another set of physically close genes, the tissue-selective expression ranking and *p* value ranking led to two different significant genes, CACNA1C and CACNA1C-AS4 respectively. CACNA1C was specifically expressed in above multiple prioritized tissues for schizophrenia (including brain-frontal cortex (BA9), brain-anterior cingulate cortex, and brain-hypothalamus). In PubMed database, there have been over 100 papers linking CACNA1C to schizophrenia (e.g., [55]). The CACNA1C-AS4 (named CACNA1C antisense RNA 4), however, had no selective expression in estimated driver tissues of schizophrenia. There are also no papers implicating this gene with schizophrenia. In addtion, there are also six genes (PPP2R2A, NGEF, KLC1, EPN2, DMTF1, and ATG13) having large selective expression score (> 900) in estimated driver tissues but having no supporting papers in PubMed, which are promising candidate susceptibility genes of this disease. Therefore, the selective expression in the estimated driver tissues is useful for discovering functionally important genes for schizophrenia.

#### Bipolar disorder (BD)

In the bipolar disorder dataset, the selective expression-based ranking led to three more conditionally significant genes, 76 vs. 73. Nineteen significant (= 25%) genes based on the selective expression ranking order in the conditional gene-based test [22] were not significant based on the statistical *p* value ranking order (Additional file 2: Table S10). However, probably because bipolar disorder is less studied than schizophrenia, none of the 19 genes had over nine PubMed hit papers in the rough in silico validation. Among the 19 genes, two had the selective-expression score over 400, CACNA1I (402.9) and LRRFIP2 (421.1). CACNA1I, encoding a subunit of calcium voltage-gated channel, has been implicated with schizophrenia [56] although it has not yet been linked to bipolar disorder. CACNA1I had four transcripts in GTEx dataset. Only two of them (ENST00000402142 and ENST00000404898) had strong selective expression in the estimated driver tissues of bipolar disorder, brain-frontal cortex (BA9) and brain-cerebellar hemisphere. LRRFIP2 encoding Leucine-rich Repeat Flightless-interacting Protein 2 had 21 transcripts in the GTEx dataset. Only two transcripts (ENST00000440742 and ENST00000487246) had selective expression in the estimated driver tissues, brain-frontal cortex (BA9) and brain-cerebellar hemisphere. Probably, because most majorities of transcripts are not selectively expressed in the brain regions, this gene was seldom studied for this brain disorder.

#### Coronary artery disease (CAD)

In the coronary artery disease dataset, the selective expression-based ranking led to five more conditionally significant genes, 48 vs. 43 (see details in Additional file 2: Table S11). Among the different significant genes, 11 significant genes according to selective expression ranking had PubMed hits, while only 6 significant genes according to the *p* value ranking had PubMed hits. For example, TCF21 had a significant conditional *p* value 9.31E−8 for association with CAD according to the tissue-selective expression ranking while it had an insignificant *p* value 0.08 according to the statistical *p* value ranking. The TCF21 was selectively expressed in multiple prioritized pathogenic tissues for CAD such as the artery coronary and adipose. Recent studies have investigated the disease mechanism of TCF21 in CAD [57, 58]. Iyer et al. proposed that TCF21 played a protective role in CAD development by inhibiting SMAD3, a central transcription factor (TF) inhibiting the cellular processes that allow smooth muscle cell (SMC) to repair the vascular lesions [57].

#### Rheumatoid arthritis (RA)

For rheumatoid arthritis, the selective expression ranking led to ~ 28% more conditionally significant genes than *p* value ranking in the conditional gene-based test (see details in Additional file 2: Table S12). Among the uniquely significant genes by different ranking, selective expression ranking detected 40 genes with PubMed hits and *p* value ranking detected only 17 such genes. For example, PTPN22 had a very significant *p* value for rheumatoid arthritis, 2.34E−121, according to the tissue-selective expression ranking while it only had a *p* value 1 according to the statistical significance ranking. The PTPN22 was selectively expressed in immune-related tissues (i.e., lymphocytes cells and spleen), and there are over 100 papers of PubMed database co-mentioning the gene and rheumatoid arthritis in the titles or abstracts. The PTPN22 acts as a negative regulator of T cell receptor (TCR), which has been suggested contributing to rheumatoid arthritis by many papers [59, 60]. However, *p* value ranking led to a physically close gene of PTPN22, RSBN1, as a significant gene (*p* = 2.59E−140) while a *p* value 1 for PTPN22. The RSBN1 was selectively expressed in the brain cerebellar hemisphere but not in RA-related tissues. We failed to find the literature supporting the role of the RSBN1 in the development of RA.

#### Total cholesterol (TC)

For total cholesterol, the selective expression ranking led to seven more conditionally significant genes than *p* value ranking in the conditional gene-based test. However, among the uniquely significant genes for the two different ranking strategies, 23 out of 54 significant genes according to selective expression ranking (~ 43%) had PubMed hits, while only 12 out of 47 significant genes according to *p* value ranking (~ 26%) have PubMed hits (see details in Additional file 2: Table S13). This suggests the selective expression ranking led not only to more significant genes but also to higher true positive rate than *p* value ranking. Here is an interesting individual example. ABCG5 and ABCG8 are physically close. The tissue-selective expression ranking made both ABCG5 and ABCG8 as candidate susceptibility genes with significant *p* values (2.56E−13 and 3.45E−25), while *p* value ranking led to an insignificant *p* value at ABCG5 (*p* = 2.8E−4). ABCG5 and ABCG8, encoding ATP-binding cassette (ABC) transporters, were selectively expressed in liver with very high significances (robust-regression *z*-scores 9955.90 and 936.422). According to literature search, over 50 papers co-mention the ABCG5/8 and total cholesterol in their titles or abstracts in PubMed database. The role of ABCG5/8 in cholesterol metabolism has been reviewed recently [61]. Actually, ABCG5 and ABCG8 form an obligate heterodimer that limits intestinal absorption and facilitate biliary secretion of cholesterol and phytosterols [61]. Hence, both ABCG5 and ABCG8 are promsing driver genes of cholesterol metabolism.

#### Height

Similarly, the selective expression ranking led to 20 more conditionally significant genes than *p* value ranking in the conditional gene-based test for height (see details in Additional file 2: Table S14). Among the uniquely significant genes detected by the two approaches, here is a representative example. The gene HFE is physically close to multi-genes encoding histone, such as HIST1H1A, HIST1H1C, HIST1H4C, and HIST1H2BC. The *p* value ranking led to a significant *p* value 1.97E−22 at HIST1H1A but an insignificant *p* value 1 at HFE. In contrast, selective expression ranking led to HFE as a candidate gene with significant *p* value (8.52E−18) and HIST1H1A as an insignificant gene (*p* = 1). The HFE was selectively expressed in the estimated driver-tissue fibroblast cells, and it regulates iron absorption by influencing the interaction of the transferrin receptor with transferrin. A study suggested the sustained enhanced iron absorption in patients with HFE hemochromatosis might have a beneficial effect on growth [62]. However, we failed to find evidence supporting the role of histone for height.

## Discussion

In the present study, we proposed a novel framework for estimating driver tissues of complex diseases and traits with gene expression and GWAS summary statistics. Using the GWAS data, this approach provides a hypothesis-free way to comprehensively explore related tissues of complex phenotypes. In the application study, it successfully detected highly related tissues consistent with known knowledge in all the six representative complex phenotypes. For instance, the brain frontal cortex (BA9) and coronary artery were ranked as the top tissues of schizophrenia and CAD respectively. More interestingly, it also suggested some cryptic driver tissues of the complex phenotypes, e.g., the adipose tissue for CAD, the lung for RA, the spleen for TC, and cardiovascular tissues for height. Some of these tissues may be not straightforward in clinical observations. Mostly, majority of the estimated tissues were validated by both independent methods and independent expression data (Fig. 3). As the expression data and GWAS summary data used in our analysis framework can be downloaded from public domains for free and have no privacy issue, the easy framework may encourage many explorations of causal tissues or cell types of complex diseases in the future, which will further facilitate molecular genetic studies and even drug target discovery [63].

Compared to two existing methods (Ongen et al. [10] and LDSC-SEG) for driver-tissue estimation, DESE has its own technique advances. First, DESE integrates the quantity of selective expression into gene-based association analysis for the estimation. Ongen et al.’s estimation was essentially built on variant-level disease association. In previous studies, we showed that gene-based association was more powerful than variant-based association [64]. This might be the reason why DESE estimated more biologically sensible tissues for almost all the tested phenotypes. Moreover, DESE also facilitates prioritizing candidate susceptibility genes, which correspondingly interprets the estimation of driver tissues. In contrast, neither of the existing tools had this important function although LDSC-SEG also extracted selectively expressed genes for inferring causal tissues. Second, DESE can directly integrate different levels of selective expression, including gene level, transcript level, and even exon level. In the present paper, we have clearly shown that a transcript (or even exon) level of selectively expression was much more powerful than the gene-level selective expression. To use the lower-level expression, Ongen et al. must calculate transcript-level or exon-level eQTLs. The substantially increased number of related transcripts and exons will complicate the analysis. By design, LDSC-SEG cannot integrate the lower level of selective expression because it did not consider the expression quantities in their analysis basically. Finally, DESE does not use any cutoffs for the selective expression, which may produce more robust estimation results. Ongen et al. needed a cutoff to select significant eQTL for driver-tissue estimation. LDSC-SEG arbitrarily selected the top 10% of genes with selective expression for driver-tissue estimation.

The observation that transcript-level selective expression is more powerful for detecting driver tissues of complex diseases than that of the gene level is biologically sensible. In cells, it is essentially the mRNA transcripts that are translated for biological functions. Different transcripts may have different functions. Therefore, the transcript-level selective expression may more precisely capture a gene’s function in specific cell types. The gene-level expression is basically an averaged expression of different transcripts, which may attenuate the tissue selectivity property of some transcripts and miss its important characteristic expressions. This may be the reason why the *p* values of estimated driver tissues based on transcript-level expression were much more significant than that based on the gene-level expression in all the proof-of-principle examples (see details in Fig. 3). These results suggest studies on transcriptome of complex diseases should pay more attention to transcript-level expression, which was often not so deeply investigated. Moreover, our results also suggest some lowly expressed transcripts may be also important for complex diseases, which led to more significant *p* values at the estimated driver tissues (see details in Table 1). The importance of these genes can be highlighted by their large selective expression values at some transcripts.

Given the importance of transcript-level selective expression, it may be tricky to select a suitable transcript representing its gene in the driver-tissue estimation. In the present study, we used the transcript with the maximal selective expression in a tested tissue for the estimation analysis. This may have bias toward genes with more transcripts as they tend to have larger selective expression by chance. However, this tendency equally occurs at every tissue. In the analysis of real examples, the usage of maximal selective expression led to the estimated driver tissues accordant with known biology of the phenotypes. There are also alternative ways of using the transcript-level selective expression. The averaged selective expression should be similar to the gene-level selective expression which led to less powerful estimation (Fig. 3). When the minimal selective expression of a gene was used, we found it almost had no power to detect significant driver tissues (Additional file 1: Table S8). Therefore, the maximal selective expression is at least an effective way for the driver-tissue estimation although it might not be the best way.

The hypothesis that genes associated with complex diseases tend to have selective expression in primary pathogenic tissues looks relatively strong. Although it has been widely accepted that causal genes of Mendelian diseases often have selective expression in the pathogenic tissues or cell types [6], it is generally unclear for complex phenotypes. The high concordance between the estimated driver tissues and known biology at all the six tested phenotypes suggested the validity of the hypothesis. It is unlikely that the high concordance in our analysis just occurred by chance. Probably, genetic perturbation in the primary causal tissues has higher functional impact on the genes with selective expression which then lead to higher impact on phenotypes. It should be noted that not all genes having selective expression in the primary pathogenic tissues are the susceptibility genes. We observed many selectively expressed genes had no significant disease-associated *p* values. A gene supported by both selective expression in the estimated driver tissues and significant phenotype-associated *p* values is more likely to be the true susceptibility gene. However, the underlying mechanism is beyond the scope of the present study.

The secondary function of DESE will also greatly facilitate genetic fine-mapping of susceptibility genes in GWAS analyses. LD is a tricky problem in GWAS for discriminating true susceptibility genes from many indirectly associated genes. Li et al. proposed a powerful statistical framework to help isolate directly associated genes [22]. However, the original analysis was carried out according to a rank of statistical significance (i.e., *p* value) assuming the true susceptibility genes had more significant *p* values. But this is not always true due to sampling fluctuations. After ranking genes according to their selective expression in the prioritized tissues for a disease, we reperformed the conditional gene-based analysis with the new rank. It turned out the selective expression ranking led to more significant genes and higher proportion of genes supported by literatures for all the six representative complex diseases/traits. These results suggest that integration of selective expression can efficiently enhance the power of identifying susceptibility genes. We believe this strategy will also work for many other complex phenotypes. It will be an effective framework to mine new susceptibility genes in the secondary analysis of GWAS data with free data in public domains.

We used four types of measures of selective expression for the driver-tissue estimation, including the proposed robust-regression *z*-score and three existing methods. This is because we found that different measures led to different significance levels in different datasets and phenotypes. For example, the proposed robust-regression *z*-score with transcript-level expression led to the highest significant *p* values for BD (Fig. 3) while the ratio of vector projection with same expression data led to more significant *p* values for schizophrenia. With the expression from GEO dataset, the proposed robust-regression *z*-score also led to the most significant *p* values at the estimated driver tissues of schizophrenia. Therefore, we used all the four measures for the estimation analysis. However, compared to the other three measures, the proposed robust-regression *z*-score has some technique advances. First, it integrates standard errors of expression means into the analysis. The variances of estimated means (i.e., standard errors) vary from tissues to tissues because of expression fluctuation and sample sizes. In GTEx dataset, for instance, the sample size of a tissue varies from 5 to 564. The means estimated in larger samples tend to be more accurate than those in smaller samples and should be given higher weights. The proposed robust-regression *z*-score extended a robust regression to subtly integrate the standard errors as weights for measuring selection expression. In contrast, existing measures can only use the estimated expression mean of each tissue for the selective expression analysis. Moreover, the proposed robust-regression *z*-score produced *p* values close to uniform distribution (Additional file 1: Figures S2 and S3). The property of being uniform greatly facilitates statistical inference of selectively expressed genes. To our knowledge, most selective expression measures (including the three ones in the present paper) cannot be used to declare selectively expressed genes by statistical *p* values.

Due to lack of data, the selective expression profiles in the collected tissues are far from complete and the developmental stages of tissues are unavailable either, which is a limitation of the present study. For example, a liver has lobes, surfaces, and impressions. In GTEx dataset, the liver has no sub-tissues. Because of this issue, some estimated driver tissues may be still rough in our analysis. Probably also due to the same reason, some genes with significant phenotype-association *p* values have no significant selective expression in the estimated driver tissues of a disease. The significance in the driver-tissue estimation analysis may be increased when expression in higher resolution tissues at suitable developmental stage is available. However, as more and more expression data are accumulating, this limitation is diminishing. The tissue-selective expression will become a powerful resource for identifying driver tissues and new susceptibility genes of human diseases.

## Methods

### A unified framework of estimating driver tissues by genes’ selective expression

The framework, named driver-tissue estimation by selective expression (DESE), consists of main three components, conditional gene-based association analysis by an effective chi-square test, estimating driver tissues according to selective expression of the conditionally associated genes, and ranking genes according to their selective expression in the estimated driver tissues. Based on the inputs of GWAS summary statistics and gene expression of multiple tissues, they run iteratively to output a converged list of driver tissues and susceptibility genes (as byproduct) with statistical *p* values (see the pipeline in Fig. 1). The following are detailed description of the three components.

#### I. Conditional gene-based association analysis with GWAS *p* values

The iterative procedure starts with production of associated genes of a disease or trait by our recent conditional gene-based association test (effective chi-square, ECS) [22] with GWAS summary statistics, which is available on the KGG platform (http://grass.cgs.hku.hk/limx/kgg/) [65]. ECS has a unique advantage of removing redundantly associated genes with the GWAS *p* values of sequence variants. The variants within upstream and downstream (say 5 kb) of a gene are assigned onto the gene according to a gene model, e.g., RefSeqGene, https://www.ncbi.nlm.nih.gov/refseq/rsg/. The ancestrally matched genotypes (e.g., Phase3 v5 Shapeit2 in 1000 Genomes Project [66]) are employed as reference genotypes for the removal of redundant association by LD in the gene-based association test. In the first iteration, genes with smaller *p* values are given higher priority to enter the conditional gene-based association analysis one by one (see details in the original paper [22]). In the following-up iteration, genes with higher tissue selectivity score are given higher priority to enter the conditional association analysis one by one (see production of the scores below). The basic assumption is that genes selectively expressed in driver tissues of a complex phenotype are more likely to be causal genes. If the true causal genes enter preferentially, the conditional gene-based association analysis will more effectively remove indirectly associated genes.

#### II. Estimate driver tissue of diseases by selective expression of disease- or trait-associated genes

Given the phenotype-associated genes from the above conditional analysis, the driver tissues of a disease are estimated by the Mann-Whitney *U* test (Wilcoxon rank-sum test) [67]. It basically tests whether the selective expression median of the phenotype-associated genes is significantly higher than that of other genes in an interrogated tissue. When a gene has multiple transcripts, the one with largest selective expression value represents the gene in the transcript-level analysis. We assume that tissue-selective expression of the associated genes determines the tissue where complex phenotype develops primarily [68]. Therefore, in the causal or driver tissue, one can observe higher selectively expression of the phenotype-associated genes. Alternatively, one can use hypergeometric distribution test to evaluate the enrichment of significant selective expression among the phenotype-associated genes. However, the enrichment results may be sensitive to the cutoff for defining significant selective expression. The Mann-Whitney *U* test has an advantage of being cutoff-free and may produce more robust results. A significant *p* value suggests the phenotype-associated genes tend to have higher selective expression in the tested tissue, indicating its potential of being a driver tissue of the corresponding disease.

#### III. Rank genes by tissue-selective expression in estimated driver tissues

The driver-tissue estimation results are then used to rank candidate genes according to their selective expression in the corresponding tissues. Denote the *p* values of above Mann-Whitney *U* test in *N* tissues as *p*_1_, ⋯, *p*_*N*_. Sort the selective expression of all genes (*T*_*i*_) in a tissue *i* in ascending order and a gene *j* is ranked at *k*_*j,i*_’s place. The gene’s selective expression score of the gene *j* in all tissues is:

$$ {\mathrm{s}}_j={\sum}_{i=1}^N\frac{k_{j,i}}{T_i}\left[-\mathrm{lo}{\mathrm{g}}_{10}\left({p}_i\right)\right]. $$

A gene having strong selective expression in multiple estimated driver tissues will obtain a high score. The ranking score is then used to determine the order in the next conditional gene-based association analysis [22], where genes with higher ranking scores will be given higher priority to enter the conditioning procedure. A gene supporting by both high-ranking score and significant phenotype-associated *p* values are highly prioritized for the phenotype as well.

The three steps are iterated until the *p* values of all tissues do not change almost. The iterative procedure for estimating driver tissues and genes has been implemented into our platform KGG, http://grass.cgs.hku.hk/limx/kgg/. See description about the implementation at Additional file 1: Text 1.

### Measures of genes’ selective expression

There have been multiple measures for evaluating the selective expression of genes [13]. Because selective expression is a relative quantity, it is often challenging to define the background reference tissues. In the present study, besides adopting three existing measures of selective expression, we also proposed a robust measure of selective expression by extending the Huber robust linear regression [69].

Let us define *N* different tissues, and each tissue has multiple transcriptome samples. A gene (or transcript) has expression means and standard errors (SE) in the *N* tissues, *y*_1_, …, *y*_*N*_ and *s*_1_, …, *s*_*N*_. Assume majority expression values approximately follow a certain distribution (say, normal distribution, or uniform distribution) while a minority of values deviate from the majority due to selective expression.

I. The proposed measure of tissue-selective expression, robust-regression *z*-score (REZ)
  We notice that the seemingly mussy values in the majority group can often approximately form a line after sorting (see illustration in Additional file 1: Figure S1). But the selective expression values will deviate from the line. In addition, as expression means of a gene in tissues with smaller SEs are often more reliable than those with larger SEs, we extended the Huber robust linear regression [69] to weight the expression deviation and reliability. The reason why we choose the Huber regression is that it is particularly efficient for outliers in the response variable than other alternative approaches [70, 71]. The regression produces smaller weights at the expression values with larger deviation from the fitted line and larger SEs:
  1 $$ \left\{\begin{array}{c}{y}_{(i)}={\beta}_0+{\beta}_1\times i+{e}_{(i)}\\ {}{w}_{(i)}=\left\{\begin{array}{c}1/{s}_{(i)}\kern1.00em \left|{e}_{(i)}\right|\le k\\ {}k/\left[{s}_{(i)}\times \left|{e}_{(i)}\right|\right]\kern0.5em \left|{e}_{(i)}\right|>k\end{array}\right.\end{array}\right. $$
  where *β*_0_ and *β*_1_ are the regression parameters and *i* ∈ [1…*N*] is the rank of a gene expression in an ascendingly sorted list of the *N* tissues. The *y*_(*i*)_ denotes the *i*th expression mean, *s*_(*i*)_ is the corresponding SE of *y*_(*i*)_, and *e*_(*i*)_ denotes the residual. When each tissue only has one subject, *y*_*i*_, is the expression value of the subject and *s*_*i*_ is set to be 1. The *w*_(*i*)_ is named a weight of *y*_(*i*)_. The *k* is a tuning constant and is equal to 1.345 **×** standard deviation of the weighted residuals [69]. The iteratively reweighted least-square procedure of robust linear regression is used to generate the converged weights of thegene at the *N* tissues, *w*_1_, …, *w*_*N*_.
  The converged weights are then standardized,
  $$ {\overset{`}{w}}_i=\frac{w_i}{\sum_{j=1}^N{w}_j}, $$
  and are used to produce a robust mean,
  $$ {\hat{\mu}}_w={\sum}_{i=1}^N{\overset{`}{w}}_i\times {y}_i $$
  and a robust standard deviation,
  $$ {\hat{\sigma}}_w=\sqrt{\frac{\sum_{i=1}^N{\overset{`}{w}}_i{\left({y}_i-{\mu}_w\right)}^2}{1-{\sum}_{i=1}^N{\overset{`}{w}}_i^2}}. $$
  The proposed robust-regression *z*-score for selective expression at tissue *i* is defined as:
  $$ {\overset{`}{z}}_i=\frac{y_i-{\hat{\mu}}_w}{{\hat{\sigma}}_w\times \lambda }. $$
  The ${\overset{`}{z}}_i$ quantifies the expression deviation from the homogenous majority expression values. The *λ* is a constant factor to adjust the *p* values to follow uniform distribution for hypothesis test. Extensive simulations suggested that an empirical factor of $\sqrt{1.5}$ led to approximately uniformly distributed *p* values (Additional file 1: Figures S2 and S3). The *p* value is then approximated based on the standard normal distribution,
  $$ {p}_i=\Big\{{\displaystyle \begin{array}{c}2\times \left[1-\varPhi \left({\overset{`}{z}}_i\right)\right],{\overset{`}{z}}_i\ge 0\\ {}2\times \varPhi \left({\overset{`}{z}}_i\right),{\overset{`}{z}}_i<0\end{array}}\operatorname{} $$, where *Φ*(*x*) is the cumulative distribution function of the standard normal distribution.

II. The conventional *z*-score for selective expression
  When tissue number is sufficiently large, the conventional *z*-score for standardization may also be effective for selective expression. The *z*-score for selective expression at a gene in tissue *i* is calculated by,
  $$ {\mathrm{z}}_{c,i}=\frac{{\mathrm{y}}_i-\hat{\mu}}{\hat{\sigma}}, $$
  where $\hat{\mu}$ is the mean of the gene expression values in all the *N* tissues and $\hat{\sigma}$ is the standard deviation.

III. Median absolute deviation (MAD) robust *z*-score
  As the mean is sensitive to outlier, the MAD robust *z*-score was also previously proposed for selective expression [72]. The median is a robust measure of central tendency and MAD is a robust measure of statistical dispersion. The MAD robust *z*-score of a gene’s selective expression in tissue *i* is estimated by:
  $$ {z}_{\mathrm{MAD},i}=\frac{\mid {\mathrm{y}}_i-M\mid }{\mathrm{MAD}}, $$
  where *M* is the expression median of the gene among the *N* tissues and MAD = 1.4826 × median(| *y*_1_ − *M*|, | *y*_2_ − *M*|, …, | *y*_*N*_ − *M*| ). Note we removed the zero values of ∣*y*_*j*_ − *M*∣ to avoid the zero MAD for the analysis.

IV. The ratio of vector-scalar projection (RVP)
  Xiao et al. proposed a ratio of vector-scalar projection for measuring selection expression of a gene in multiple tissues [16]. The RVP of a gene’s selective expression in tissue *i* is estimated by:
  $$ {\mathrm{RVP}}_i={y}_i^2/{\sum}_{j=1}^N{y}_j^2. $$
  The RVP ranges from 0 to 1.0. A value close to 1.0 indicates that tissue *i* is the major contributor to the projected length of vector *Y* = (*y*_1_, *y*_2_, …, *y*_*N*_) in high-dimension tissue spaces or, biologically, high tissue selectivity.

### Gene expression datasets and quality control

We adopted three independent expression datasets for driver-tissue estimation and validation. The calculated and QC-pass expression means and SE of these datasets are available for download at http://grass.cgs.hku.hk/limx/rez/.

#### The RNA-Seq profiles from GTEx project

The normalized expression datasets at the gene level and transcript level were downloaded from GTEx project (V7) [73], GTEx_Analysis_2016-01-15_v7_RNASeQCv1.1.8_gene_tpm.gct.gz and GTEx_Analysis_2016-01-15_v7_RSEMv1.2.22_transcript_tpm.txt.gz. The sample sizes of each tissue were different, ranging from 5 to 564 (Additional file 1: Table S2). There were initially 196,520 transcripts and 56,205 genes in 53 tissues. The expression values were measured by transcripts per kilobase million (TPM). As TPM is effective for cross-tissue comparison [74], we did not retransform the expression values by other measurements. A series of quality control procedures were carried out. The mean and standard deviation of expression values of all genes in each tissue were calculated. In the evaluation according to correlation, three tissues (the whole blood, pancreas, and pituitary) had low Pearson correlation with other tissues (Fig. 2a and Additional file 1: Figure S4) and were excluded. In the calculation of tissue-selective expression, genes or transcripts having ≤ 0.01 TPM in all tissues were excluded. Genes whose Ensembl IDs had no corresponding official HGNC gene symbols were excluded as well. Finally, 131,292 transcripts and 31,659 genes in 50 tissues were retained for subsequent analysis.

#### The microarray expression profiles from Gene Expression Omnibus (GEO) repository

We also curated microarray expression profiles of multiple tissues from GEO database for validation. We adopted the tissue-tree structure in Open Biomedical Ontologies (https://www.ebi.ac.uk/ols/ontologies/bto) to collect the expression data. Stringent quality controls were carried out in the expression data. For each individual subject, the expression values of genes were ranked. The gene expression values were standardized corresponding to the quantile of the genes in the ranking list under standard normal distribution. This transformation converted data to normal distribution and eliminated systematic batch effects. Within each GEO-GSE dataset of the same tissue, we removed samples which had relatively low and high correction with majority samples according to the Pearson correlation coefficients. The correlations below 5% of all correlations and above 95% correlations were defined as the low and the high correlation respectively. We explored the GEO database and retrieved GSE datasets according to tissue-tree structure. If a tissue had over 200 subjects, its offspring tissues were checked. If one or more offspring tissues also had the sample size over 200, the exploration went further into the offspring of the offspring tissues. Otherwise, the exploration stopped at the current tissue node with 200 or more subjects. In total, 55 different tissues were collected from the GEO database. Additional file 1: Table S3 lists the tissue names and the corresponding sample sizes. Genes with 9 or few expression values were removed. Finally, 19,012 genes were retained.

#### The RNA-Seq profiles from BrainSpan

We also downloaded gene- and exon-level transcription profiles from BrainSpan as an independent dataset for validation analysis. According to our previous study, the expression in prenatal brains may be less effective for illustrating disease association based on common variants [22]. We therefore removed expression profiles of prenatal brains. Genes or exons with 9 or few expression values were removed. Finally, 16 brain regions were retained for the driver-tissue estimation analysis. The number of genes and exons were 34,172 and 187,184 respectively. The region names and sample sizes are listed in Additional file 1: Table S4.

### Produce genes associated with six representative complex diseases or traits

We collected the GWAS meta-analysis *p* values at single nucleotide polymorphisms (SNPs) of six representative complex diseases or traits developing in different biological systems, schizophrenia [23], bipolar disorder [26] (brain diseases), rheumatoid arthritis (RA) [75] (an autoimmune disease), coronary artery disease (CAD) [76] (a cardiovascular disease), total cholesterol [77] (a metabolic trait), and height [46] (an anthropometric trait). Additional file 1: Table S6 lists the sample sizes and downloading links of the datasets. The *p* values of SNPs were combined for gene-based association on KGG (see detailed methods in the above section). The significantly associated genes (after multiple testing correction) were used to detect potential driver tissues and to finely map susceptibility genes by the conditional gene-based test [22] (see pipeline in Fig. 1).

### Validate and compare estimated driver tissues by the proposed framework with existing methods

#### The method of Ongen et al.

Ongen et al. estimated the driver tissues of a complex disease based on concordance of active expression quantitative trait loci (eQTLs) and GWAS-associated variants [10]. They assumed the causal tissue showed significantly higher concordance than irrelevant tissues, which is different from the hypothesis of DESE. Because Ongen et al.’s method had no publicly available tools, we directly extracted enrichment values from the Supplementary Table S5 of their published paper for the validation and performance comparison [10]. According to Ongen et al., the tissues with the enrichment value over the null greater than 1 were considered as the significant causal tissues for the diseases/traits.

#### LDSC-SEG

Finucane et al. proposed to infer causal tissues according to heritability enrichment at selectively expressed genes [11], which was named LD score regression applied to specifically expressed genes (LDSC-SEG). They adopted a *t*-statistic to evaluate selective expression of genes for a focal tissue. The top 10% of genes according to large *t*-statistic were chosen as specifically expressed gene set. Sequence variants within 100 kb upstream and downstream of selectively expressed genes were included for calculating heritability by stratified LD score regression. The selectively expressed genes were assumed to contribute higher heritability in causal tissues than in irrelevant tissues. Therefore, the enriched heritability was used to infer causal tissues of complex diseases in turn. Their estimated driver tissues were successfully validated by chromatin data. In the present study, we directly adopted the results in the Supplementary Table 6 (their main results) and the Supplementary Table 7 (chromatin validation results) of Finucane et al. [11] for the validation and comparison.

### In silico validation by PubMed search

We used PubMed search function to roughly validate the detected genes for a complex disease. The underlying assumption is that a gene’s contribution to a disease can be indicated by the observation that multiple papers co-mention the gene and the disease name in the title or abstract. The more involved papers, the more likelihood the gene is related to the disease. Although this may be crude for one individual gene, it can produce a reliable evaluation when there are many genes. We employed the web application programming interfaces (APIs) of PubMed to execute the search. The search link was http://eutils.ncbi.nlm.nih.gov/entrez/eutils/esearch.fcgi?db=pubmed&term=“DiseaseNames (inlcuding homonymies)”[tiab]%29 + AND+“GeneSymbol (including RefSeq mRNA IDs)” [tiab]. The search results included PubMed ID and relevant data of the papers, if available, in extensible markup language (XML).

## Additional files

Additional file 1: Supplementary text, figures and tables (PDF 1711 kb)

Additional file 2: Supplementary Excel tables (XLSX 233 kb)

## Data Availability

The KGG (V4.1) is available from http://grass.cgs.hku.hk/limx/kgg/. Furthermore, the source code of KGG (V4.1) can be downloaded from Github (https://github.com/pmglab/KGG) [78] and Zenodo (10.5281/zenodo.3367790) [79]. The code is released under MIT license (https://opensource.org/licenses/MIT). The robust selective expression of genes from public datasets including GTEx (V7) and GEO can be queried and downloaded from http://grass.cgs.hku.hk/limx/rez. The expression dataset of GTEx was obtained from dbGaP (accession number phs000424.v2.p1), BrainSpan from http://www.brainspan.org (prior to October 2013), and GEO from https://www.ncbi.nlm.nih.gov/geo (prior to October 2017).
