# Supplementary material for: DESE: estimating driver tissues by selective expression of genes associated with complex diseases or traits
Source: Genome Biol. 2019 Nov 6;20:233. doi: 10.1186/s13059-019-1801-5 (PMC6836538; doi:10.1186/s13059-019-1801-5)
Supplement: Supplementary file 1 — Supplementary text, figures and tables (PDF 1711 kb) [file 13059_2019_1801_MOESM1_ESM.pdf]

Supplementary Text:

1. Implementation

We have implemented and released DESE into a user-friendly software platform KGG (http://grass.cgs.hku.hk/limx/kgg/) for estimating driver tissues and prioritizing susceptibility genes. KGG is a standalone tool encoded by Java. Users input GWAS summary statistics and expression values of multiple tissues. The tool will calculate selective expression of genes by four different measures and will then estimate driver tissues based on the selective expression of phenotype-associated genes by the iterative procedure. The phenotype-associated genes were calculated by gene-based association analysis from the GWAS summary statistics. In addition, users can input genes through a web browser to query specific expression at transcripts in multiple tissues. See details of the tool on the webpage, http://grass.cgs.hku.hk/limx/rez/.

Supplementary Figures

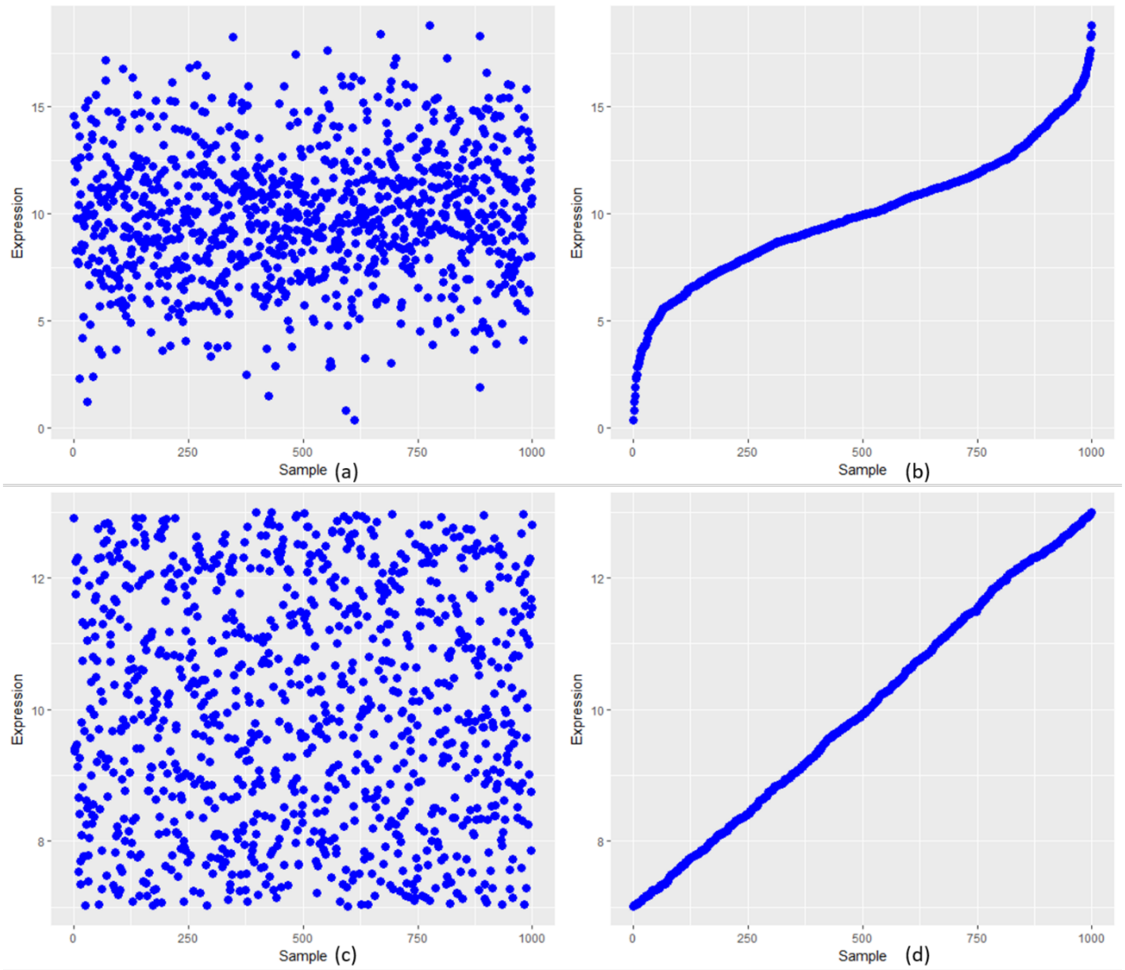

Figure S1: Illustration of approximate linear features of normalized expression values after sorting. a) the expression values prior to sorting under normal distribution b) the expression values after sorting under normal distribution, c) the expression values prior to sorting under uniform distribution d) the expression values after sorting under uniform distribution

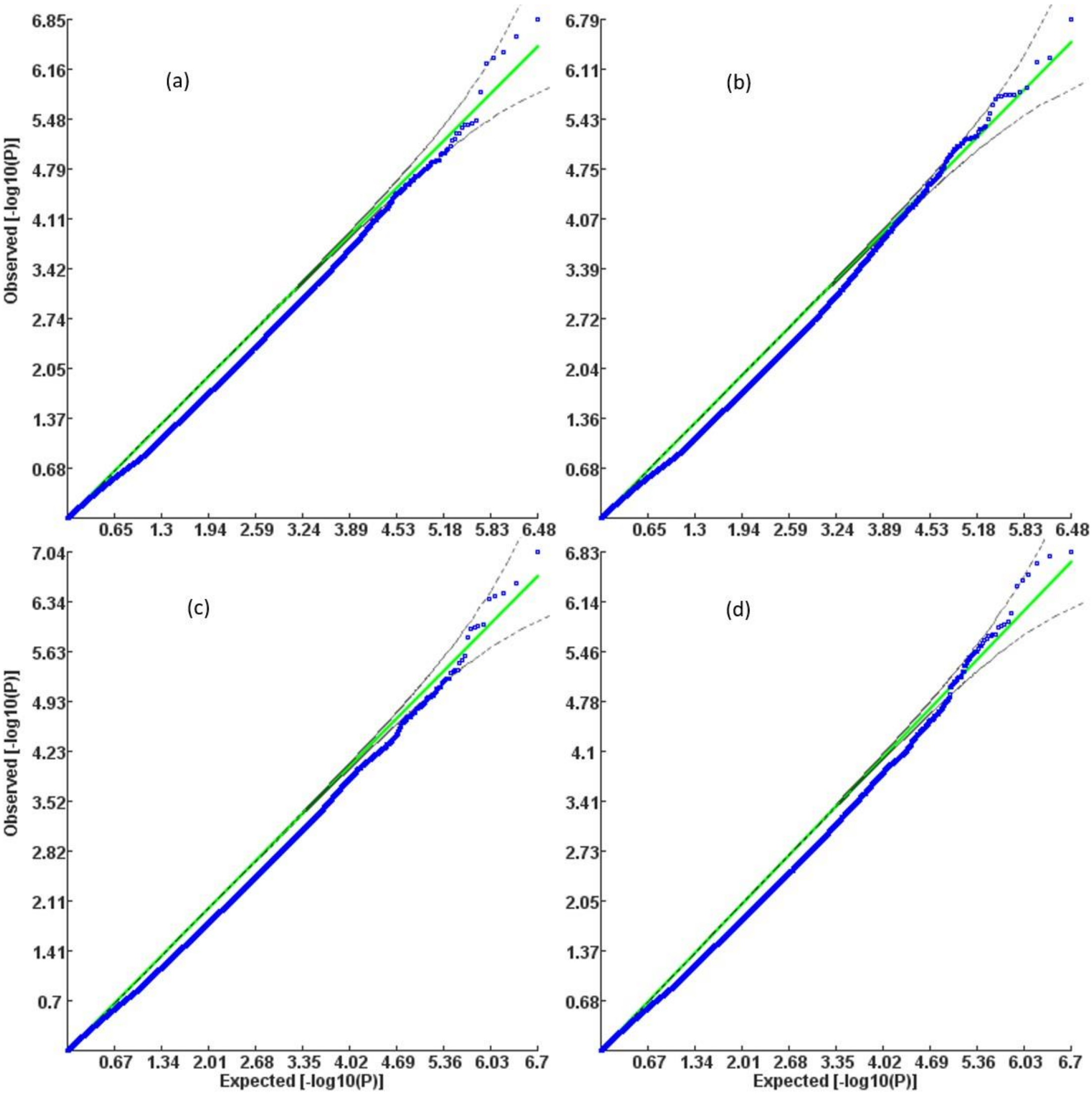

Figure S2: QQ plot of for detecting tissue-specific expression under simulated normally distributed expression values a)the distribution of expression: N(1,1), number of tissues:30, and sample size of each tissue [70,100]; b)the distribution of expression: N(10,5), number of tissues:30, and sample size of each tissue [70,100]; c)the distribution of expression: N(10,10), number of tissues:50, and sample size of each tissue [70,100]; d)the distribution of expression: N(10,10), number of tissues:50, and sample size of each tissue [300,400]. In each scenario, 100,000 datasets were simulated for the analysis.

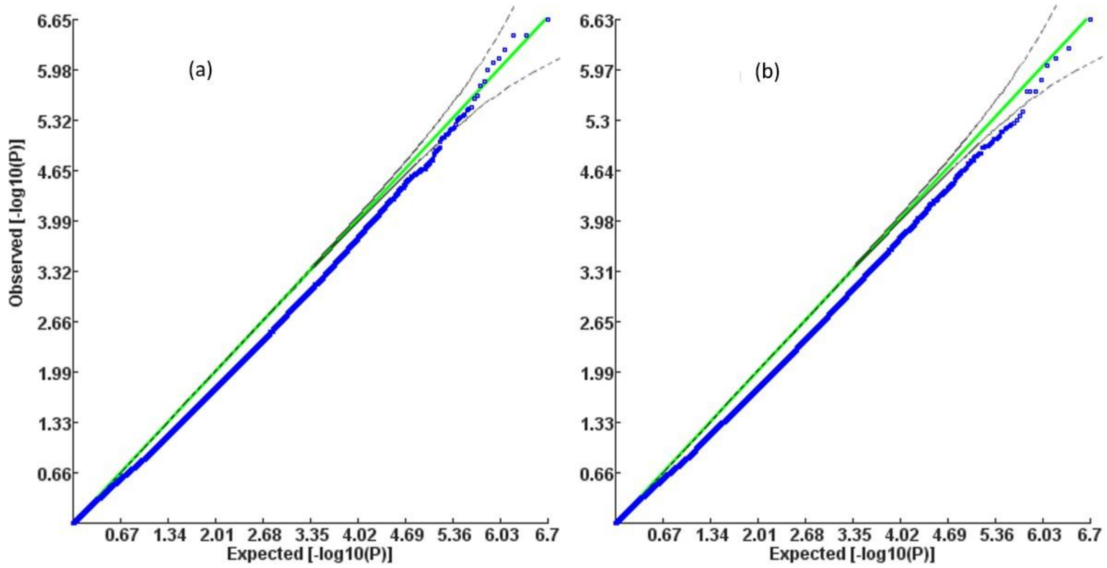

Figure S3: QQ plot of for detecting tissue-specific expression under simulated uniformly distributed expression values a)the distribution of expression: U(5,15), number of tissues:50, and sample size of each tissue [70,100]; b)the distribution of expression: U(25,75), number of tissues:50, and sample size of each tissue [300,400]. In each scenario, 100,000 datasets were simulated for the analysis.

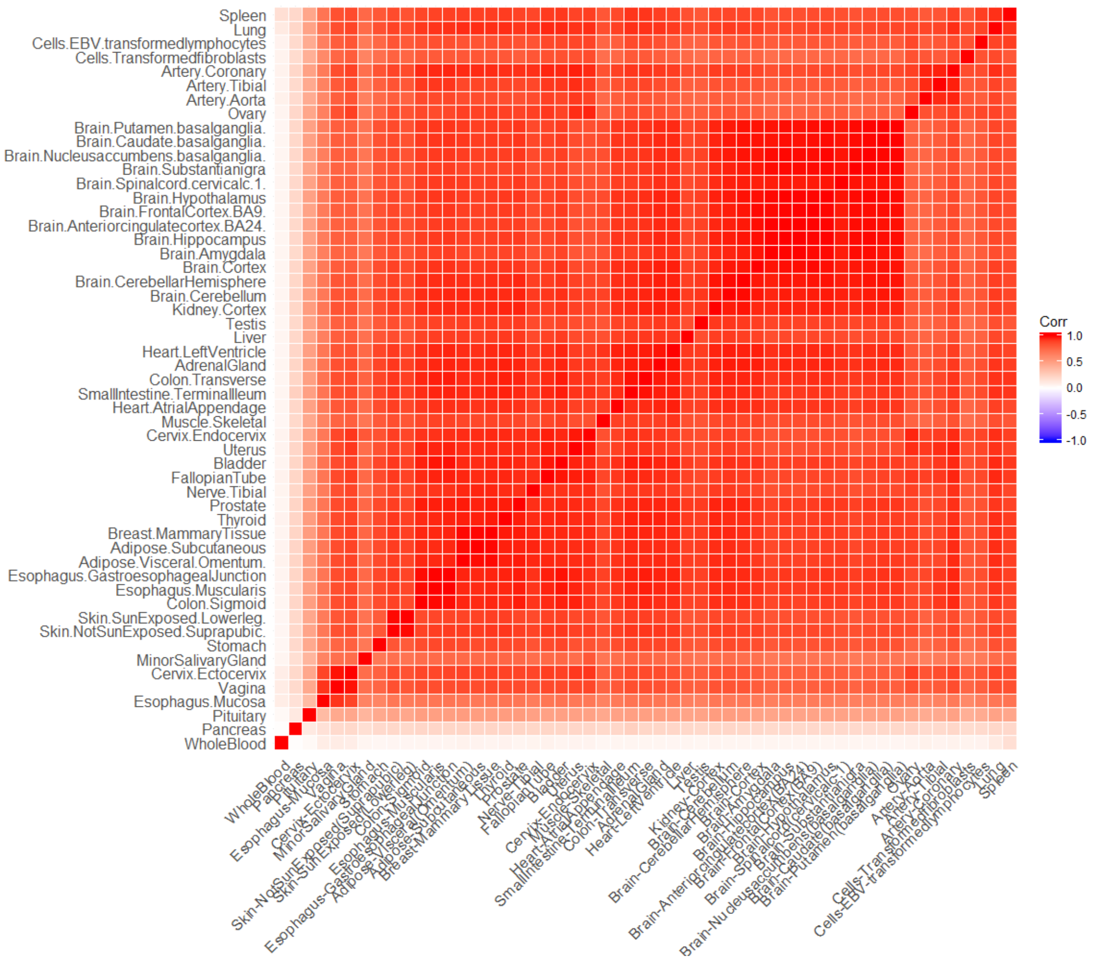

Figure S4: Pearson correlation of the tissues according to the TPM of expression values at genes.

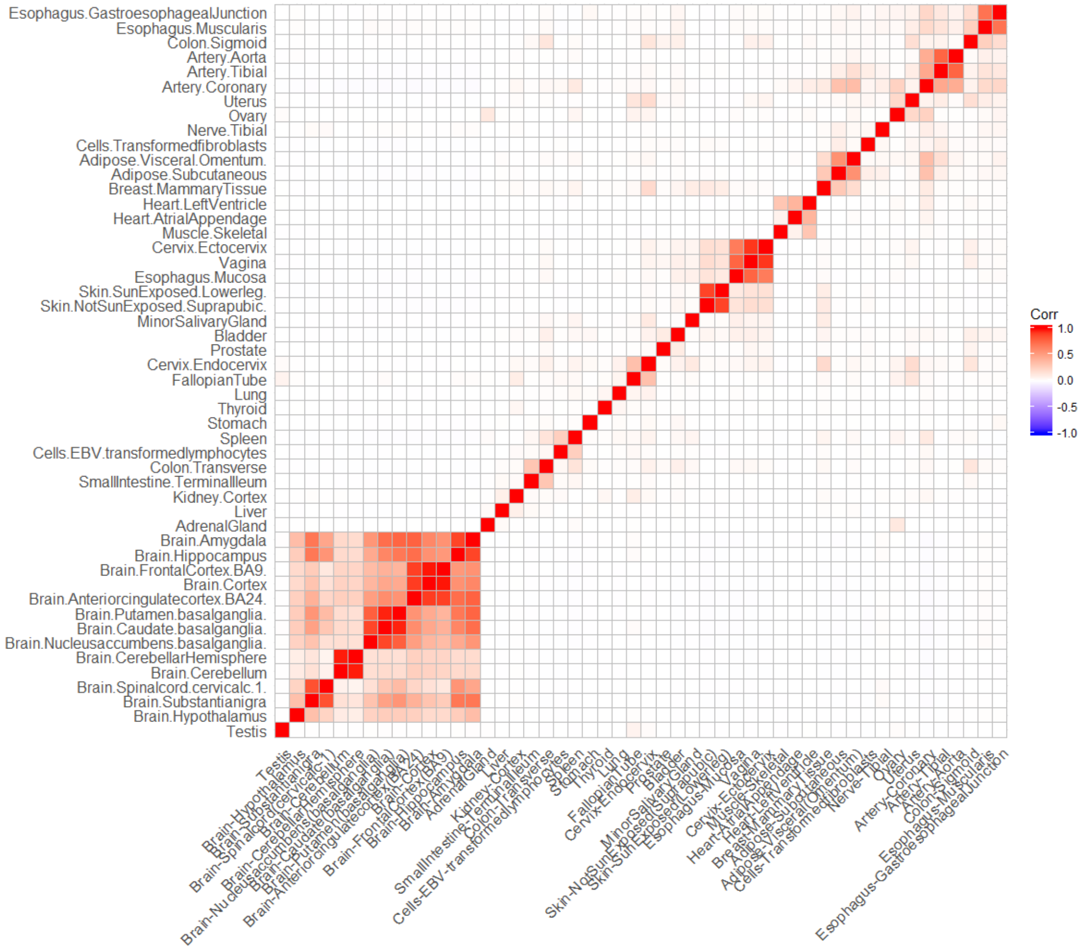

Figure S5: Pearson correlation of the tissues calculated by the robust-z scores at genes.

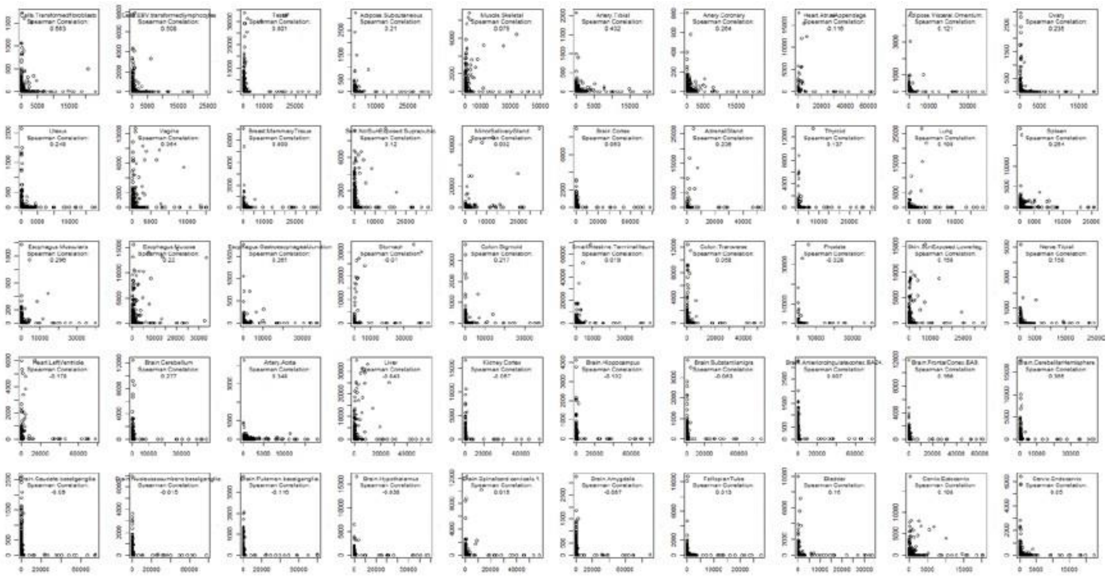

Figure S6: The scatter plots and correlation of original expression score and tissue specific scores at gene levels. The horizontal axis denotes the TPM score and vertical axis denotes the robust z-scores.

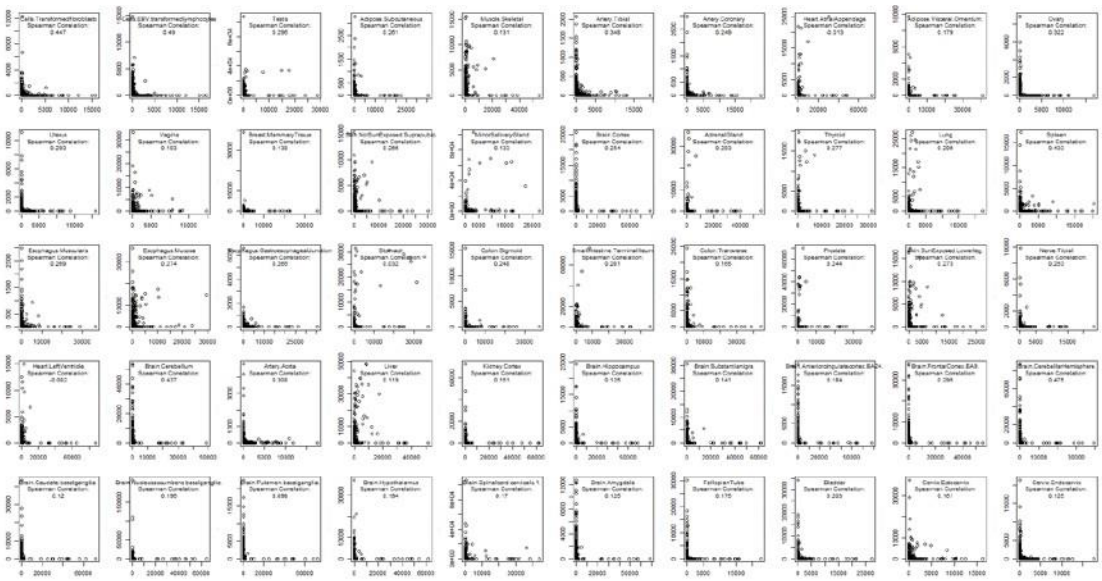

Figure S7: The scatter plots and correlation of original expression score and tissue specific expression scores at transcript levels. The horizontal axis denotes the TPM score and vertical axis denotes the robust z-scores.

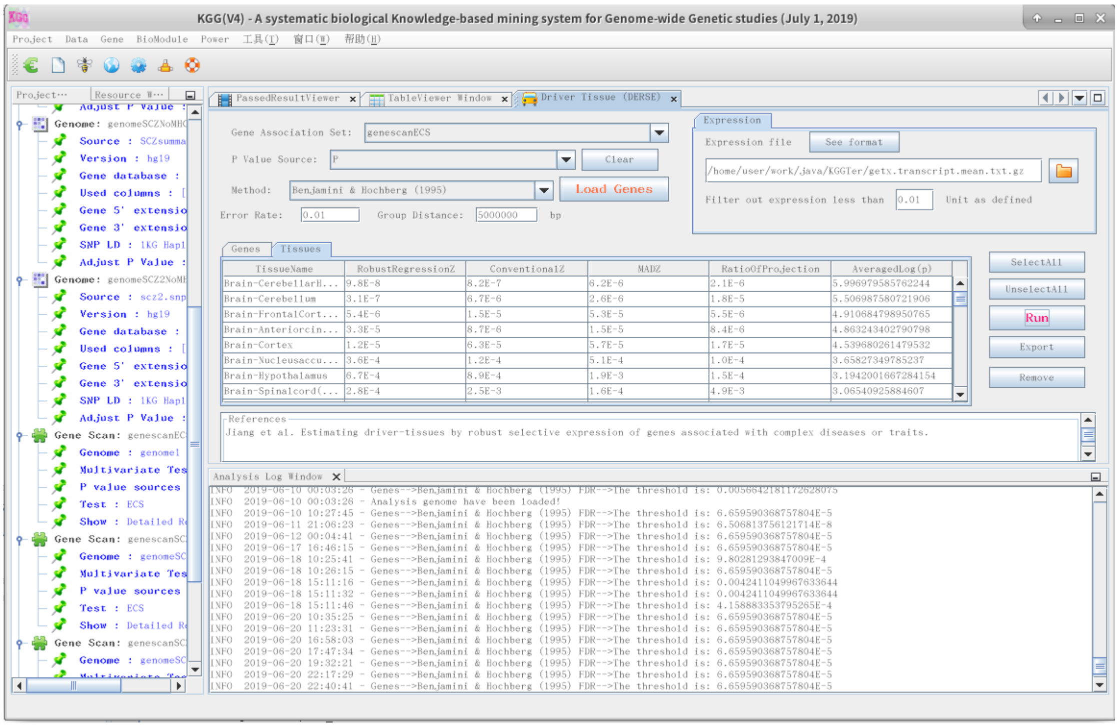

Figure S8: The graphic interface of KGG for driver-tissue estimation by DESE, On the dialog, users can set p-value threshold to extract significant phenotype-associatied genes, the minimal distance to set gene groups for conditional gene-based association analysis, and set minimal expression values to exclude genes or transcripts with too low expression.

Supplementary Tables

Table S1: Statistical power of the robust z-score approach and standard z-score approach

Number selectively expressed tissues	Expression value Deviated from mean (SD)	Power of the robust-regression z-score(%)	Power of the conventional z-score(%)	Number selectively expressed tissues	Expression value Deviated from mean (SD)	Power of the robust-regression z-score (%)	Power of the conventional z-score(%)
1	1	100.0	100.0	1	-1	100.0	100.0
	2	100.0	100.0		-2	100.0	100.0
	3	100.0	100.0		-3	100.0	100.0
3	1	99.9	78.8	3	-1	100.0	78.7
	2	100.0	99.7		-2	100.0	99.7
	3	100.0	100.0		-3	100.0	100.0
6	1	80.4	0.5	6	-1	80.3	0.5
	2	99.9	0.0		-2	99.9	0.0
	3	100.0	0.0		-3	100.0	0.0

Note: Fifty expression values were generated under a normal distribution with mean 10 and standard deviation 10, *N*(10, 10). Each power is estimated by 100,000 simulated datasets.

Table S2: Sample sizes of different tissues from GTEx

Tissue/CellType	Size
Adipose-Subcutaneous	442
Adipose-Visceral(Omentum)	355
AdrenalGland	190
Artery-Aorta	299
Artery-Coronary	173
Artery-Tibial	441
Bladder	11
Brain-Amygdala	100
Brain-Anteriorcingulatecortex(BA24)	121
Brain-Caudate(basalganglia)	160
Brain-CerebellarHemisphere	136
Brain-Cerebellum	173
Brain-Cortex	158
Brain-FrontalCortex(BA9)	129
Brain-Hippocampus	123
Brain-Hypothalamus	121
Brain-Nucleusaccumbens(basalganglia)	147
Brain-Putamen(basalganglia)	124
Brain-Spinalcord(cervicalc-1)	91
Brain-Substantianigra	88
Breast-MammaryTissue	290
Cells-EBV-transformedlymphocytes	130
Cells-Transformedfibroblasts	343
Cervix-Ectocervix	6
Cervix-Endocervix	5
Colon-Sigmoid	233
Colon-Transverse	274
Esophagus-GastroesophagealJunction	244
Esophagus-Mucosa	407
Esophagus-Muscularis	370
FallopianTube	7
Heart-AtrialAppendage	297
Heart-LeftVentricle	303
Kidney-Cortex	45
Liver	175
Lung	427
MinorSalivaryGland	97
Muscle-Skeletal	564
Nerve-Tibial	414
Ovary	133
Pancreas	248
Pituitary	183
Prostate	152

**Skin-NotSunExposed(Suprapubic)**	387
**Skin-SunExposed(Lowerleg)**	473
**SmallIntestine-TerminalIleum**	137
**Spleen**	162
**Stomach**	262
**Testis**	259
**Thyroid**	446
**Uterus**	111
**Vagina**	115
**WholeBlood**	407

Table S3: Sample sizes of different tissue from GEO database

Tissue name	Sample size
**myocardium.BTO_0000901**	929
**left-ventricle.BTO_0001629**	978
**endothelial-cell.BTO_0001176**	336
**vein.BTO_0000234**	313
**aorta.BTO_0000135**	266
**coronary-artery.BTO_0000290**	403
**capillary.BTO_0002045**	634
**embryonic-stem-cell.BTO_0001086**	213
**umbilical-cord.BTO_0001415**	1093
**retina.BTO_0001175**	347
**choroid.BTO_0001829**	305
**nasal-mucosa.BTO_0000912**	247
**bronchial-epithelial-cell.BTO_0002922**	204
**substantia-nigra.BTO_0000143**	309
**hypophysis.BTO_0001073**	359
**amygdala.BTO_0001042**	270
**prefrontal-cortex.BTO_0002807**	680
**superior-frontal-gyrus.BTO_0004836**	257
**parietal-lobe.BTO_0001001**	360
**hippocampus.BTO_0000601**	677
**cerebral-gyrus.BTO_0002495**	458
**glial-cell.BTO_0002606**	206
**cerebral-gray-matter.BTO_0000823**	215
**lateral-ventricle.BTO_0000879**	226
**tooth.BTO_0000397**	265
**osteoblast.BTO_0001593**	204
**cancellous-bone.BTO_0001700**	222
**skin-fibroblast.BTO_0001255**	222
**synovium.BTO_0001823**	250
**adipocyte.BTO_0000443**	266
**subcutaneous-adipose-tissue.BTO_0004042**	1502

**breast.BTO_0000149**	730
**kidney.BTO_0000671**	2265
**prostate–gland.BTO_0001129**	230
**hepatocyte.BTO_0000575**	338
**pancreas.BTO_0000988**	227
**lymph–node.BTO_0000784**	374
**blast–cell.BTO_0000125**	878
**blood–plasma.BTO_0000131**	2489
**lymphocyte.BTO_0000775**	214
**tonsil.BTO_0001387**	281
**epithelial–cell.BTO_0000414**	291
**intestinal–mucosa.BTO_0000642**	209
**uterine–endometrium.BTO_0001422**	471
**keratinocyte.BTO_0000667**	386
**vastus–lateralis.BTO_0001563**	1910
**hamstring–muscle.BTO_0003179**	218
**smooth–muscle.BTO_0001260**	574
**ileum.BTO_0000620**	625
**colon.BTO_0000269**	1080
**knee.BTO_0003595**	334
**hand.BTO_0004668**	432
**prepuce.BTO_0001113**	697
**uterine–cervix.BTO_0001421**	327
**oocyte.BTO_0000964**	323

Table S4: Sample sizes of different brain regions from BrainSpan database

**Regions**	**Abbreviation**	**Sample Size**
**inferolateral temporal cortex (area TEv, area 20)**	ITC	13
**primary somatosensory cortex (area S1, areas 3,1,2)**	S1C	11
**posteroventral (inferior) parietal cortex**	IPC	13
**primary visual cortex (striate cortex, area V1/17)**	V1C	11
**posterior (caudal) superior temporal cortex (area 22c)**	STC	13
**mediodorsal nucleus of thalamus**	MD	9
**ventrolateral prefrontal cortex**	VFC	12
**hippocampus (hippocampal formation)**	HIP	12
**amygdaloid complex**	AMY	12
**primary motor cortex (area M1, area 4)**	M1C	11
**cerebellar cortex**	CBC	13
**dorsolateral prefrontal cortex**	DFC	11
**striatum**	STR	9

**primary auditory cortex (core)**	A1C	12
**anterior (rostral) cingulate (medial prefrontal) cortex**	MFC	11
**orbital frontal cortex**	OFC	11

Table S5: Number of significantly expressed genes according to the robust z-scores at transcript

Tissue/Cell Type	By gene expression	By transcript expression	Overlapped significant genes	Uniquely significant by gene expression	Uniquely significant by transcript expression
**Adipose-Subcutaneous**	1864	3185	1507	357	1678
**Adipose-Visceral(Omentum)**	2107	3358	1675	432	1683
**AdrenalGland**	1539	3226	1245	294	1981
**Artery-Aorta**	1899	3522	1504	395	2018
**Artery-Coronary**	1842	2977	1437	405	1540
**Artery-Tibial**	1611	3701	1302	309	2399
**Bladder**	3413	8278	2427	986	5851
**Brain-Amygdala**	2348	4983	1867	481	3116
**Brain-Anteriorcingulatecortex(BA24)**	2685	5813	2126	559	3687
**Brain-Caudate(basalganglia)**	2682	5584	2110	572	3474
**Brain-CerebellarHemisphere**	4910	12133	3742	1168	8391
**Brain-Cerebellum**	5073	12302	3788	1285	8514
**Brain-Cortex**	3104	6519	2425	679	4094
**Brain-FrontalCortex(BA9)**	3287	7205	2629	658	4576
**Brain-Hippocampus**	2525	5365	2037	488	3328
**Brain-Hypothalamus**	3113	6413	2469	644	3944
**Brain-Nucleusaccumbens(basalganglia)**	2972	6346	2330	642	4016
**Brain-Putamen(basalganglia)**	2221	4701	1781	440	2920
**Brain-Spinalcord(cervicalc-1)**	2834	5717	2199	635	3518
**Brain-Substantianigra**	2343	4824	1885	458	2939
**Breast-MammaryTissue**	2543	3834	1893	650	1941
**Cells-EBV-transformedlymphocytes**	3765	8080	2876	889	5204
**Cells-Transformedfibroblasts**	2278	6923	1801	477	5122
**Cervix-Ectocervix**	3717	8457	2560	1157	5897
**Cervix-Endocervix**	4174	9917	2780	1394	7137
**Colon-Sigmoid**	1592	2683	1314	278	1369
**Colon-Transverse**	2429	3727	1929	500	1798
**Esophagus-GastroesophagealJunction**	1136	2037	971	165	1066
**Esophagus-Mucosa**	2459	4173	1958	501	2215
**Esophagus-Muscularis**	1064	2111	921	143	1190
**FallopianTube**	4491	10142	3062	1429	7080
**Heart-AtrialAppendage**	1027	2322	840	187	1482
**Heart-LeftVentricle**	675	1785	549	126	1236
**Kidney-Cortex**	2328	4378	1773	555	2605
**Liver**	1922	3698	1536	386	2162
**Lung**	4107	6807	3162	945	3645
**MinorSalivaryGland**	2825	4597	2125	700	2472

**Muscle-Skeletal**	1170	3365	973	197	2392
**Nerve-Tibial**	2841	5033	2056	785	2977
**Ovary**	2945	4360	1811	1134	2549
**Prostate**	3181	5430	2449	732	2981
**Skin-NotSunExposed(Suprapubic)**	2760	4811	2184	576	2627
**Skin-SunExposed(Lowerleg)**	2656	4829	2140	516	2689
**SmallIntestine-TerminalIleum**	3394	5489	2686	708	2803
**Spleen**	3917	6456	2938	979	3518
**Stomach**	2002	2790	1487	515	1303
**Testis**	12520	26086	8911	3609	17175
**Thyroid**	3382	5766	2387	995	3379
**Uterus**	2147	3806	1510	637	2296
**Vagina**	2920	4688	2219	701	2469

Note: The Bonferroni correction was used to control family-wise error rate of 0.05 on the entire genome for declaring significantly expressed genes. When using z-score at transcripts, the cutoff for a gene was further divided by the number of transcripts according to the Bonferroni correction.

Table S6: GWAS summary results information of six complex diseases/traits

Trait name (short)	Sample size	Marker counts	Web link
Schizophrenia	94,490	4,303,605	http://gwas.bio-x.cn/datasets/summary.gz
Bipolar disorder	74,194	7,882,596	https://www.med.unc.edu/pgc/results-and-downloads
Rheumatoid arthritis (RA)	103,638	9,739,303	https://grasp.nhlbi.nih.gov/downloads/ResultsOctober2016/Okada/RA_GWASmeta_TransEthnic_v2.txt.gz
Coronary artery disease	184,305	9,446,655	http://www.cardiogramplusc4d.org/media/cardiogramplusc4d-consortium/data-downloads/cad.additive.Oct2015.pub.zip
Total cholesterol (TC)	188,577	2,446,981	https://grasp.nhlbi.nih.gov/downloads/ResultsOctober2016/Global_Lipids_Genetics_Consortium/jointGwasMc_TC.txt.gz
Height	253,288	2,547,478	https://grasp.nhlbi.nih.gov/downloads/ResultsOctober2016/Wood/GIANT_HEIGHT_Wood_et_al_2014_publicrelease_HapMapCeuFreq.txt.gz

Table S7: Estimation with transcript level selective expression and original expression from GTEx

Schizophrenia			Bipolar disorder		
**TissueName**	**Proposed robust**	**Original expression**	**TissueName**	**Proposed robust Z**	**Original expression**
**Brain-Anteriorcingulatecortex(BA24)**	5.30E-13	3.60E-10	**Brain-CerebellarHemisphere**	9.8E-08	5E-06
**Brain-FrontalCortex(BA9)**	5.30E-13	1.70E-10	**Brain-Cerebellum**	3.1E-07	2E-05
**Brain-Cortex**	1.80E-12	3.60E-10	**Brain-FrontalCortex(BA9)**	5.4E-06	7E-06
**Brain-Nucleusaccumbens(basalganglia)**	1.10E-11	4.50E-09	**Brain-Anteriorcingulatecortex(BA24)**	0.000033	2E-05
**Brain-Putamen(basalganglia)**	6.50E-11	6.70E-09	**Brain-Cortex**	0.000012	1E-05
**Brain-Caudate(basalganglia)**	8.70E-11	9.70E-09	**Brain-Nucleusaccumbens(basalganglia)**	0.00036	1E-04
**Brain-Hippocampus**	4.40E-11	3.60E-09	**Brain-Hypothalamus**	0.00067	0.0001
**Brain-Amygdala**	1.50E-11	9.60E-09	**Brain-Caudate(basalganglia)**	0.0025	0.0001
**Brain-Hypothalamus**	1.80E-10	1.3E-08	**Brain-Spinalcord(cervi**	0.00028	0.0053

			calc-1)		
**Brain-Substantianigra**	8.60E-11	9.7E-08	**Brain-Putamen(basalganglia)**	0.0021	0.0004
**Brain-Spinalcord(cervicalc-1)**	0.000000024	0.000001	**Brain-Amygdala**	0.0017	0.0004
**Esophagus-GastroesophagealJunction**	0.000000016	0.000068	**Colon-Sigmoid**	0.0018	0.0026
**Esophagus-Muscularis**	0.00000039	0.000084	**Brain-Hippocampus**	0.0018	0.0003
**Brain-Cerebellum**	0.0000093	2.4E-06	**Heart-AtrialAppendage**	0.00031	0.019
**Artery-Tibial**	0.0000054	0.00022	**Brain-Substantianigra**	0.013	0.001
**Colon-Sigmoid**	0.000002	0.00013	**Colon-Transverse**	0.0033	0.019
**Brain-CerebellarHemisphere**	0.000031	4.9E-06	**Heart-LeftVentricle**	0.0015	0.021
**Heart-AtrialAppendage**	0.000032	0.00065	**AdrenalGland**	0.00064	0.017
**Nerve-Tibial**	0.00017	0.0002	**Esophagus-Muscularis**	0.0042	0.013
**Artery-Aorta**	0.00019	0.00062	**Artery-Tibial**	0.0031	0.016
**Artery-Coronary**	0.000022	0.00095	**Cells-Transformedfibroblasts**	0.0021	0.047
**Ovary**	0.00032	0.00038	**Uterus**	0.018	0.0092
**Vagina**	0.00035	0.002	**Esophagus-GastroesophagealJunction**	0.01	0.01
**AdrenalGland**	0.00027	0.00096	**Adipose-Visceral(Omentum)**	0.0038	0.073
**Adipose-Subcutaneous**	0.001	0.0017	**Bladder**	0.01	0.06
**Uterus**	0.00078	0.0006	**Testis**	0.014	0.035
**Muscle-Skeletal**	0.00035	0.0045	**Cervix-Endocervix**	0.013	0.045
**Testis**	0.0033	0.00091	**Artery-Aorta**	0.028	0.042
**Adipose-Visceral(Omentum)**	0.00084	0.0012	**Stomach**	0.027	0.045
**Cervix-Endocervix**	0.005	0.014	**Prostate**	0.051	0.022
**Skin-SunExposed(Lowerleg)**	0.008	0.0031	**Artery-Coronary**	0.026	0.03
**Bladder**	0.0022	0.018	**Vagina**	0.0083	0.024
**Heart-LeftVentricle**	0.002	0.01	**Liver**	0.058	0.034
**Cells-Transformedfibroblasts**	0.0063	0.012	**FallopianTube**	0.034	0.04
**Breast-MammaryTissue**	0.025	0.0018	**Muscle-Skeletal**	0.071	0.11
**Skin-NotSunExposed(Suprapubic)**	0.021	0.0057	**MinorSalivaryGland**	0.029	0.12
**Prostate**	0.034	0.0044	**Ovary**	0.11	0.022
**Cervix-Ectocervix**	0.0099	0.012	**Kidney-Cortex**	0.094	0.099
**Colon-Transverse**	0.003	0.0018	**Breast-MammaryTissue**	0.055	0.069
**Stomach**	0.0043	0.0038	**Adipose-Subcutaneous**	0.052	0.059
**Thyroid**	0.04	0.0034	**SmallIntestine-TerminalIleum**	0.05	0.051
**Kidney-Cortex**	0.028	0.015	**Esophagus-Mucosa**	0.059	0.14
**FallopianTube**	0.038	0.019	**Skin-SunExposed(Lowerleg)**	0.12	0.074
**Esophagus-Mucosa**	0.047	0.015	**Spleen**	0.073	0.094
**Lung**	0.13	0.011	**Nerve-Tibial**	0.18	0.023
**Cells-EBV-transformedlymphocytes**	0.024	0.05	**Skin-NotSunExposed(Suprapubic)**	0.19	0.12
**SmallIntestine-TerminalIleum**	0.18	0.0091	**Lung**	0.15	0.072

**Liver**	0.12	0.032	**Cervix-Ectocervix**	0.093	0.077
**MinorSalivaryGland**	0.13	0.036	**Cells-EBV-transformedlymphocytes**	0.12	0.36
**Spleen**	0.9	0.043	**Thyroid**	0.35	0.11

Table S8: Estimation with maximal and minimal transcript-level selective expression

Schizophrenia			Bipolar disorder		
**Tissue Name**	maximal	minimal	**TissueName**	maximal	minimal
**Brain-FrontalCortex(BA9)**	5.3E-13	0.67	**Brain-CerebellarHemisphere**	1.3E-09	0.38
**Brain-Anteriorcingulatecortex(BA24)**	5.3E-13	0.67	**Brain-Cerebellum**	7.3E-09	0.38
**Brain-Cortex**	1.8E-12	0.87	**Brain-FrontalCortex(BA9)**	1.6E-06	0.43
**Brain-Nucleusaccumbens(basalganglia)**	1.1E-11	0.67	**Brain-Cortex**	0.000017	0.53
**Brain-Amygdala**	1.5E-11	0.8	**Brain-Anteriorcingulatecortex(BA24)**	0.000024	0.48
**Brain-Hippocampus**	4.4E-11	0.69	**Heart-AtrialAppendage**	0.000038	0.66
**Brain-Putamen(basalganglia)**	6.5E-11	0.49	**AdrenalGland**	0.000058	0.78
**Brain-Substantianigra**	8.6E-11	0.5	**Brain-Nucleusaccumbens(basalganglia)**	0.00012	0.89
**Brain-Caudate(basalganglia)**	8.7E-11	0.43	**Brain-Hippocampus**	0.00028	0.79
**Brain-Hypothalamus**	1.8E-10	0.67	**Brain-Spinalcord(cervicalc-1)**	0.00031	0.97
**Esophagus-GastroesophagealJunction**	1.6E-08	1	**Brain-Hypothalamus**	0.00033	0.89
**Brain-Spinalcord(cervicalc-1)**	2.4E-08	0.95	**Heart-LeftVentricle**	0.00039	0.74
**Esophagus-Muscularis**	3.9E-07	1	**Colon-Transverse**	0.0005	0.92
**Colon-Sigmoid**	0.000002	0.99	**Cells-Transformedfibroblasts**	0.00052	0.97
**Artery-Tibial**	5.4E-06	0.75	**Artery-Tibial**	0.00059	0.82
**Brain-Cerebellum**	9.3E-06	1	**Esophagus-Muscularis**	0.00063	0.96
**Artery-Coronary**	0.000022	1	**Adipose-Visceral(Omentum)**	0.00068	0.94
**Brain-CerebellarHemisphere**	0.000031	1	**Colon-Sigmoid**	0.00084	0.86
**Heart-AtrialAppendage**	0.000032	0.94	**Brain-Amygdala**	0.0011	0.8
**Nerve-Tibial**	0.00017	1	**Brain-Putamen(basalganglia)**	0.0013	0.91
**Artery-Aorta**	0.00019	0.97	**Brain-Caudate(basalganglia)**	0.0015	0.91
**AdrenalGland**	0.00027	0.99	**Adipose-Subcutaneous**	0.0027	0.99
**Ovary**	0.00032	0.94	**Esophagus-GastroesophagealJunction**	0.003	0.93
**Vagina**	0.00035	0.99	**Artery-Coronary**	0.0057	1
**Muscle-Skeletal**	0.00035	0.99	**Vagina**	0.0061	0.99
**Uterus**	0.00078	0.99	**Artery-Aorta**	0.0098	1
**Adipose-Visceral(Omentum)**	0.00084	0.98	**Prostate**	0.0098	0.99
**Adipose-Subcutaneous**	0.001	0.99	**Stomach**	0.0099	0.9
**Heart-LeftVentricle**	0.002	0.99	**Uterus**	0.01	0.97
**Bladder**	0.0022	1	**Esophagus-Mucosa**	0.01	0.98
**Colon-Transverse**	0.003	1	**Breast-MammaryTissue**	0.011	0.98
**Testis**	0.0033	1	**Lung**	0.012	0.99
**Stomach**	0.0043	0.99	**Skin-SunExposed(Lowerleg)**	0.013	0.95
**Cervix-Endocervix**	0.005	0.96	**Brain-Substantianigra**	0.014	0.88
**Cells-Transformedfibroblasts**	0.0063	1	**MinorSalivaryGland**	0.015	0.99
**Skin-SunExposed(Lowerleg)**	0.008	0.96	**SmallIntestine-TerminalIleum**	0.017	0.99
**Cervix-Ectocervix**	0.0099	0.99	**Muscle-Skeletal**	0.021	0.92
**Skin-NotSunExposed(Suprapubic)**	0.021	0.99	**Bladder**	0.022	0.97

**Cells-EBV-transformedlymphocytes**	0.024	1	**Testis**	0.032	0.97
**Breast-MammaryTissue**	0.025	1	**FallopianTube**	0.036	0.95
**Kidney-Cortex**	0.028	1	**Ovary**	0.04	1
**Prostate**	0.034	1	**Skin-NotSunExposed(Suprapubic)**	0.041	0.97
**FallopianTube**	0.038	1	**Spleen**	0.044	0.98
**Thyroid**	0.04	1	**Nerve-Tibial**	0.049	1
**Esophagus-Mucosa**	0.047	1	**Cervix-Endocervix**	0.052	0.85
**Liver**	0.12	1	**Cells-EBV-transformedlymphocytes**	0.059	1
**MinorSalivaryGland**	0.13	1	**Liver**	0.06	0.96
**Lung**	0.13	1	**Thyroid**	0.063	1
**SmallIntestine-TerminalIleum**	0.18	1	**Cervix-Ectocervix**	0.1	0.99
**Spleen**	0.9	1	**Kidney-Cortex**	0.11	0.99
